# Deep Metabolomic Profiling Reveals Alterations in Fatty Acid Synthesis and Ketone Body Degradations in Spermatozoa and Seminal Plasma of Astheno-Oligozoospermic Bulls

**DOI:** 10.3389/fvets.2021.755560

**Published:** 2022-01-11

**Authors:** Mohua Dasgupta, Arumugam Kumaresan, Kaustubh Kishor Saraf, Pradeep Nag, Manish Kumar Sinha, Muhammad Aslam M. K., Gayathree Karthikkeyan, T. S. Keshava Prasad, Prashant Kumar Modi, Tirtha Kumar Datta, Kerekoppa Ramesha, Ayyasamy Manimaran, Sakthivel Jeyakumar

**Affiliations:** ^1^Theriogenology Laboratory, Southern Regional Station of Indian Council of Agricultural Research (ICAR)—National Dairy Research Institute, Bengaluru, India; ^2^Base Farm, Kerala Veterinary and Animal Sciences University, Kolahalamedu, India; ^3^Centre for Systems Biology and Molecular Medicine, Yenepoya Research Centre, Yenepoya (Deemed to be University), Mangalore, India; ^4^Animal Genomics Laboratory, Indian Council of Agricultural Research (ICAR)—National Dairy Research Institute, Karnal, India; ^5^Dairy Production Section, Southern Regional Station of Indian Council of Agricultural Research (ICAR)—National Dairy Research Institute, Bengaluru, India

**Keywords:** metabolomics, spermatozoa, seminal plasma, dairy bulls, mass spectrometry, semen quality

## Abstract

Male fertility is extremely important in dairy animals because semen from a single bull is used to inseminate several thousand females. Asthenozoospermia (reduced sperm motility) and oligozoospermia (reduced sperm concentration) are the two important reasons cited for idiopathic infertility in crossbred bulls; however, the etiology remains elusive. In this study, using a non-targeted liquid chromatography with tandem mass spectrometry-based approach, we carried out a deep metabolomic analysis of spermatozoa and seminal plasma derived from normozoospermic and astheno-oligozoospermic bulls. Using bioinformatics tools, alterations in metabolites and metabolic pathways between normozoospermia and astheno-oligozoospermia were elucidated. A total of 299 and 167 metabolites in spermatozoa and 183 and 147 metabolites in seminal plasma were detected in astheno-oligozoospermic and normozoospermic bulls, respectively. Among the mapped metabolites, 75 sperm metabolites were common to both the groups, whereas 166 and 50 sperm metabolites were unique to astheno-oligozoospermic and normozoospermic bulls, respectively. Similarly, 86 metabolites were common to both the groups, whereas 45 and 37 seminal plasma metabolites were unique to astheno-oligozoospermic and normozoospermic bulls, respectively. Among the differentially expressed metabolites, 62 sperm metabolites and 56 seminal plasma metabolites were significantly dysregulated in astheno-oligozoospermic bulls. In spermatozoa, selenocysteine, deoxyuridine triphosphate, and nitroprusside showed significant enrichment in astheno-oligozoospermic bulls. In seminal plasma, malonic acid, 5-diphosphoinositol pentakisphosphate, D-cysteine, and nicotinamide adenine dinucleotide phosphate were significantly upregulated, whereas tetradecanoyl-CoA was significantly downregulated in the astheno-oligozoospermia. Spermatozoa from astheno-oligozoospermic bulls showed alterations in the metabolism of fatty acid and fatty acid elongation in mitochondria pathways, whereas seminal plasma from astheno-oligozoospermic bulls showed alterations in synthesis and degradation of ketone bodies, pyruvate metabolism, and inositol phosphate metabolism pathways. The present study revealed vital information related to semen metabolomic differences between astheno-oligozoospermic and normospermic crossbred breeding bulls. It is inferred that fatty acid synthesis and ketone body degradations are altered in the spermatozoa and seminal plasma of astheno-oligozoospermic crossbred bulls. These results open up new avenues for further research, and current findings can be applied for the modulation of identified pathways to restore sperm motility and concentration in astheno-oligozoospermic bulls.

## Introduction

Infertility is a multifactorial problem; a majority of infertility cases are idiopathic and molecular exploration on spermatozoa, and seminal plasma revealed that 50% of such problems were associated with a male partner (1). Male fertility assumes much significance in dairy animals because semen from a single male is used to inseminate several thousand females. To improve milk production, crossbreeding of native cattle with exotic milch breeds is being taken up in several countries. Although crossbreeding helps in the genetic improvement of the offspring in milk production, a considerable proportion of male offspring suffer from infertility/subfertility. It is well-documented that the magnitude of infertility is higher in crossbred bulls as compared with purebred bulls; ~40–70% of crossbred males are culled every year due to subfertility/infertility (2–4). Several studies reported that asthenozoospermia (reduced sperm motility), oligozoospermia (reduced sperm concentration), and poor cryotolerance are the major reasons for idiopathic infertility in crossbred bulls leading to high rates of culling among crossbred breeding bulls (5–8). Basically, astheno-oligozoospermia condition is often associated with incomplete spermiogenesis and teratological defects of sperm, impeding cervical mucus penetration and reaching the site of fertilization (9). Immature spermatocytes in astheno-oligozoospermia patients appear as a good source of reactive oxygen species (ROS) production, which alters mitochondrial DNA, membrane potential, depletes adenosine triphosphate, and triggers extensive damage to the lipid-rich membrane of spermatid and mature spermatozoa (10). All these could lead to DNA damage in the normal sperm population and reduced fertility because semen with higher fragmented DNA is associated with male infertility (11).

Recent advances in “OMICS” techniques helped in expanding our knowledge and understanding of astheno-oligozoospermia at the transcriptomic and proteomic levels in human beings (12, 13); however, the etiology still remains elusive. Apart from the defects in spermatogenesis, astheno-oligozoospermia condition is related to the role of epididymis and accessory glands secretions as well (14). At ejaculation, epididymal spermatozoa are diluted with the secretions from various accessory sex glands (an admixture of various proteins, hormones, and metabolites) that regulate sperm motility and functions (15). Metabolites, being the end product of any biological process, regulate the downstream events of gene expression (16), and their entity within spermatozoa may be sugar, lipid, amino acid, nucleoside, mineral, vitamins, and nucleoside (17). Recent pieces of evidence indicate metabolomic alteration in the Krebs cycle and energy metabolism pathways in spermatozoa and seminal plasma from human astheno-oligozoospermia patients (17–19). Distinct divergence of metabolic profile between high-fertile and low-fertile bull spermatozoa (4, 20) and seminal plasma (21) has also been reported. Therefore, any alteration at the metabolome level may be one of the sublimed reasons behind the idiopathic astheno-oligozoospermia condition in a crossbred bull. Thus, the study of sperm and seminal fluid composition in conditions associated with infertility could help in improving diagnostic accuracy and also lead to a better understanding of the pathophysiology of infertility (22).

Although metabolome of human spermatozoa in relation to specific conditions are available, information on the metabolomic profile of bull sperm and seminal plasma is very limited, and metabolomic alterations in spermatozoa and seminal plasma from astheno-oligozoospermic bulls are not available. We hypothesized that specific metabolic pathways might be altered in astheno-oligozoospermic spermatozoa and seminal fluid. Hence, the study aimed to (i) analyze the metabolomic composition of spermatozoa and seminal plasma in normozoospermia bulls and (ii) identify the spermatozoa and seminal plasma metabolomic alterations in astheno-oligozoospermic bulls. In the present study, we applied a liquid chromatography with tandem mass spectrometry (LC-MS/MS)-based approach to identify the metabolomic alterations in spermatozoa and seminal plasma from astheno-oligozoospermic bulls as compared with normozoospermia bulls. Furthermore, the functional importance of sperm and seminal plasma metabolites in bull fertility was also elucidated using bioinformatics tools. To the best of our knowledge, this is the first study comparing sperm and seminal plasma metabolites between astheno-oligozoospermic and normozoospermic bulls.

## Materials and Methods

### Ethical Statement

The present study was conducted at the Theriogenology Laboratory, Southern Regional Station of Indian Council of Agricultural Research (ICAR)—National Dairy Research Institute, Bengaluru, India, and was duly approved by the Animal Ethics Committee of ICAR—National Dairy Research Institute (CPCSEA/IAEC/LA/SRS-ICAR-NDRI-2017/No.09) and conducted as per the guidelines.

### Classification of Bulls and Sample Preparation

Semen samples collected from normozoospermic and astheno-oligozoospermic bulls were used for metabolomic analysis. For classification of the bulls, semen production data from crossbred bulls (*n* = 60; Holstein Friesian crossbred) were analyzed over some time. Ejaculates were collected using the artificial vagina method as per the standard procedure and were analyzed for sperm concentration and motility. The bulls consistently produced ejaculates with ≥600 million spermatozoa/ml and with ≥70% motile spermatozoa were categorized into the normozoospermia group, whereas bulls consistently produced ejaculates with ≤400 million spermatozoa/ml and with ≤40% motile spermatozoa were categorized into the astheno-oligozoospermia group. Ejaculates were collected from normozoospermic and astheno-oligozoospermic bulls (*n* = 3 representative bulls in each category; three ejaculates from each bull) and used for metabolomic analysis. Both the sperm concentration and motility were significantly (*P* < 0.01) lower in astheno-oligozoospermic bulls as compared with normozoospermic bulls (Supplementary Figure 1).

After collection, fresh ejaculates were centrifuged (700×*g*, 4°C, 10 min) to separate the seminal plasma and spermatozoa. Immediately after removing the seminal plasma, spermatozoa were purified following the procedure given by Parthipan et al. (23). Briefly, spermatozoa pellet was purified using 90–45% discontinuous Percoll gradient centrifugation by adding 0.9 ml of 45% of Percoll fraction in phosphate-buffered saline (PBS) over 0.2 ml of 90% fraction in 1.5-ml Eppendorf tube (Axygen, USA). The spermatozoa pellet dissolved in PBS was carefully layered over the top of the prepared Percoll gradient fraction and centrifuged at 800×*g* for 15 min at room temperature. The purified sperm pellet was washed thrice using PBS (137-mM sodium chloride; 2.7-mM potassium chloride; 10-mM sodium phosphate dibasic; 1.8-mM potassium dihydrogen phosphate; pH 7.4) at the same speed. The purified spermatozoa pellet and seminal plasma were stored in −80°C until further processing.

### Metabolite Extraction

Lysis of spermatozoa was carried out using QSonica Probe sonicator at 20% amplitude with a pulse on for 30 s and off for 5 s. Protein concentrations in sperm lysate and seminal plasma were estimated by bicinchoninic acid assay (Thermo Scientific, USA). Sample normalization was carried out using protein concentrations across samples, and an equal quantity of normalized samples from each bull was pooled to make one sample each for the normozoospermia and astheno-oligozoospermia groups. Briefly, 100 μg of protein equivalent sample was taken for metabolite extraction. Extraction was performed using 2:2:1 of acetonitrile–methanol–water. Extraction solutions were vortexed at room temperature for 1 min and sonicated in a water bath for 10 min. After sonication, the sample was centrifuged at 12,000×*g* for 15 min at 4°C. The supernatant was collected in a new tube and processed for drying in speedVac for 1 h (Thermo Scientific) and stored in −80°C until further proceeding for LC-MS/MS analysis.

### Liquid Chromatography With Tandem Mass Spectrometry Analysis

QTRAP 6500 mass spectrometer, AB Sciex, was coupled with Agilent 1290 Infinity II liquid chromatography system, with C18 RRHD Zorbax column (20 × 150 mm, 1.8-μm particle size) as the analytical column. Analyst software version 1.6.3 with the Analyst Device Driver was used to set the parameters for the analysis. The separation of the metabolites was carried out using a 30-min LC method. Solvent A was 0.1% formic acid in MilliQ water, and solvent B was 0.1% formic acid in 90% acetonitrile; the flow rate was set to 0.3 ml/min. The LC method was *t* = 0 min, 2% B; *t* = 10, 30% B; *t* = 17, 60% B; *t* = 22–26, 95% B and *t* = 27.5–30, 2% B. The MS data acquisition was carried out with the information-dependent acquisition method in low mass mode. The information-dependent acquisition method was built using the enhanced mass spectra to enhanced product ion modes. The top five spectra from the enhanced mass spectra mode were used for analysis in the enhanced product ion (MS/MS) mode, using high-energy collision-induced dissociation, i.e., collisionally induced dissociation. The metabolite data were acquired in both positive and negative polarities at 4,500 and −4,500 V, respectively, with a probe temperature of 450°C. The compound parameters were set at a declustering potential of 75 V and collision energy of 45 V. The data were acquired in triplicates, and intermediate blank runs were injected after every triplicate run of the samples to prevent carryover.

### Data Analysis

LC-MS/MS raw data format “.wiff” files from Analyst software were converted to “.mzML” using MSConvert (24). Data processing was carried out using iMETQ software (25). Peak detection was carried out with mzwidth = 0.03 and noise level = 3, and peak alignment was carried out with mzTol = 0.03 Da and rtTol = 0.16 min. The aligned peak list across samples were used for metabolite identification using Human Metabolome DataBase (HMDB, www.hmdb.ca) (26). Metabolite search was carried out with a precursor mass error of 0.05 Da, adducts based on charge state, i.e., (M+H)+, (M+2H)2+, and (M+3H)3+ and (M-H)−, (M-2H)2−, and (M-3H)3−; the identifications with the low mass error were considered for further analysis. Metaboanalyst tool (www.metaboanalyst.ca) was used for statistical and pathway analyses. This tool offers a variety of methods. In univariate analysis methods, fold change analysis, *T*-tests, volcano plot test, and correlation test analysis are used with *p*-values adjusted to 0.05, based on which significantly altered metabolites were selected. On the other hand, in multivariate analysis, partial least-squares discriminant analysis (PLSDA) and principal component analysis and, in partial clustering analysis, *K*-means clustering were used. However, before statistical analysis, data were normalized to quantile normalization, log transformation, and pareto-scaled, which minimizes the difference among the samples.

### Analysis of Metabolic Network

Metscape version 3.2.1, a plugin for Cytoscape version 3.2.1, was used for the analysis of the most abundant metabolites with the highest variable importance in projection (VIP) scores in sperm and seminal plasma of astheno-oligozoospermia and normozoospermia bulls.

## Results

### Metabolomic Profile of Spermatozoa and Seminal Plasma

Using LC-MS/MS technique, we detected 1,902 (1,323 in positive mode; 579 in negative mode) and 1,141 (537 in positive mode; 604 in negative mode) peaks in spermatozoa of astheno-oligozoospermic and normozoospermic bulls, respectively. Of which, 299 and 167 metabolites were found with Kegg IDs and PubChem IDs in HMDB search in spermatozoa of astheno-oligozoospermic and normozoospermic bulls, respectively. Similarly, 1,082 (435 in positive mode; 647 in negative mode) peaks were detected in seminal plasma of astheno-oligozoospermic bulls, whereas 988 (373 in positive mode; 615 in negative mode) peaks were detected in seminal plasma of normozoospermic bulls. A total of 183 and 147 metabolites were found with Kegg IDs and PubChem IDs in HMDB search in seminal plasma of astheno-oligozoospermic and normozoospermic bulls, respectively.

**Figure 1:**
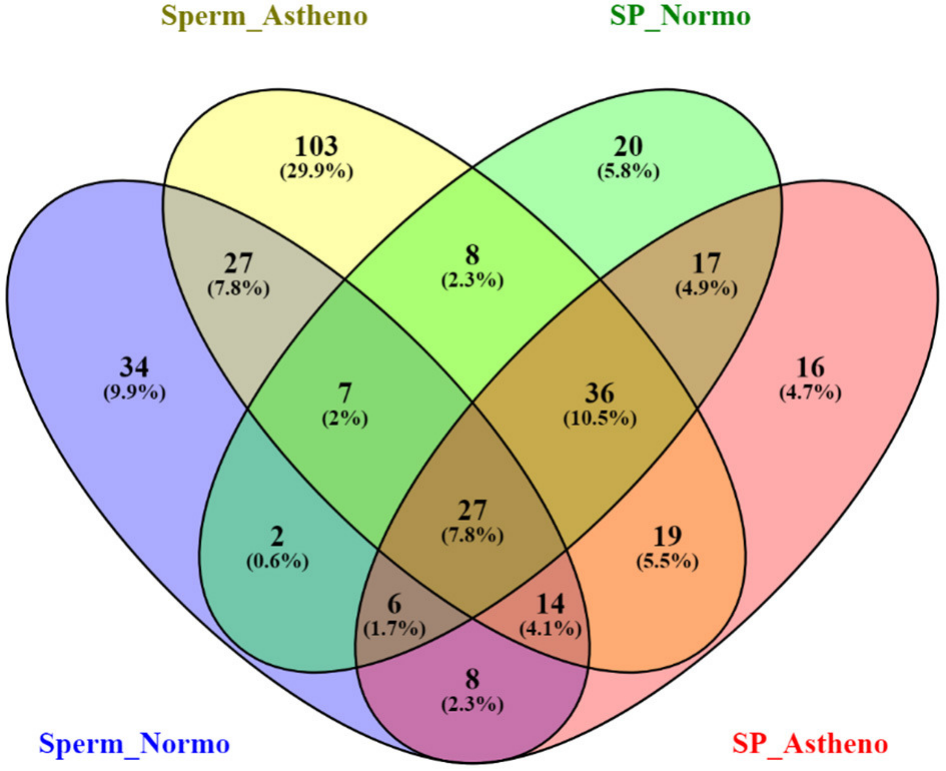
Venn diagram of total mapped metabolites of spermatozoa and seminal plasma of astheno-oligozoospermia and normozoospermia bulls in HMDB search.

After sorting out the metabolite IDs, the majority of the identified endogenous metabolites are categorized into lipid, phospholipid, glycerophospholipid, amines, common amino acid, fats, and common fatty acid based on functional enrichment of chemical compound in MBrole 2.0 (http://csbg.cnb.csic.es/mbrole2). Antibiotics, pesticides, and heavy metals were also identified as exogenous metabolites in MBrole 2.0, which were excluded from further downstream analysis. Regardless of its polarities, sorted endogenous metabolites of both groups were consolidated to carry out the statistical analysis and enrichment of metabolic pathways in MetaboAnalyst software. LC/MS-MS chromatogram of some important peaks of endogenous metabolites of spermatozoa and seminal plasma of both the groups are represented in Supplementary Figures 2A,B as documentation of stable and reliable analytical method. The Venn diagram (Figure 1) represents the total mapped metabolites, where 75 metabolites were common to both the groups of spermatozoa, whereas 166 and 50 putative spermatozoal metabolites were unique to astheno-oligozoospermic and normozoospermic bulls, respectively. Similarly, 86 metabolites were common to both the groups of seminal plasma, whereas 45 and 37 putative seminal plasma metabolites were unique to astheno-oligozoospermic and normozoospermic bulls, respectively (Venn diagram drawing tool 2.1.0. (http://bioinfogp.cnb.csic.es/tools/venny/).

### Differentially Expressed Metabolites

**Figure 2:**
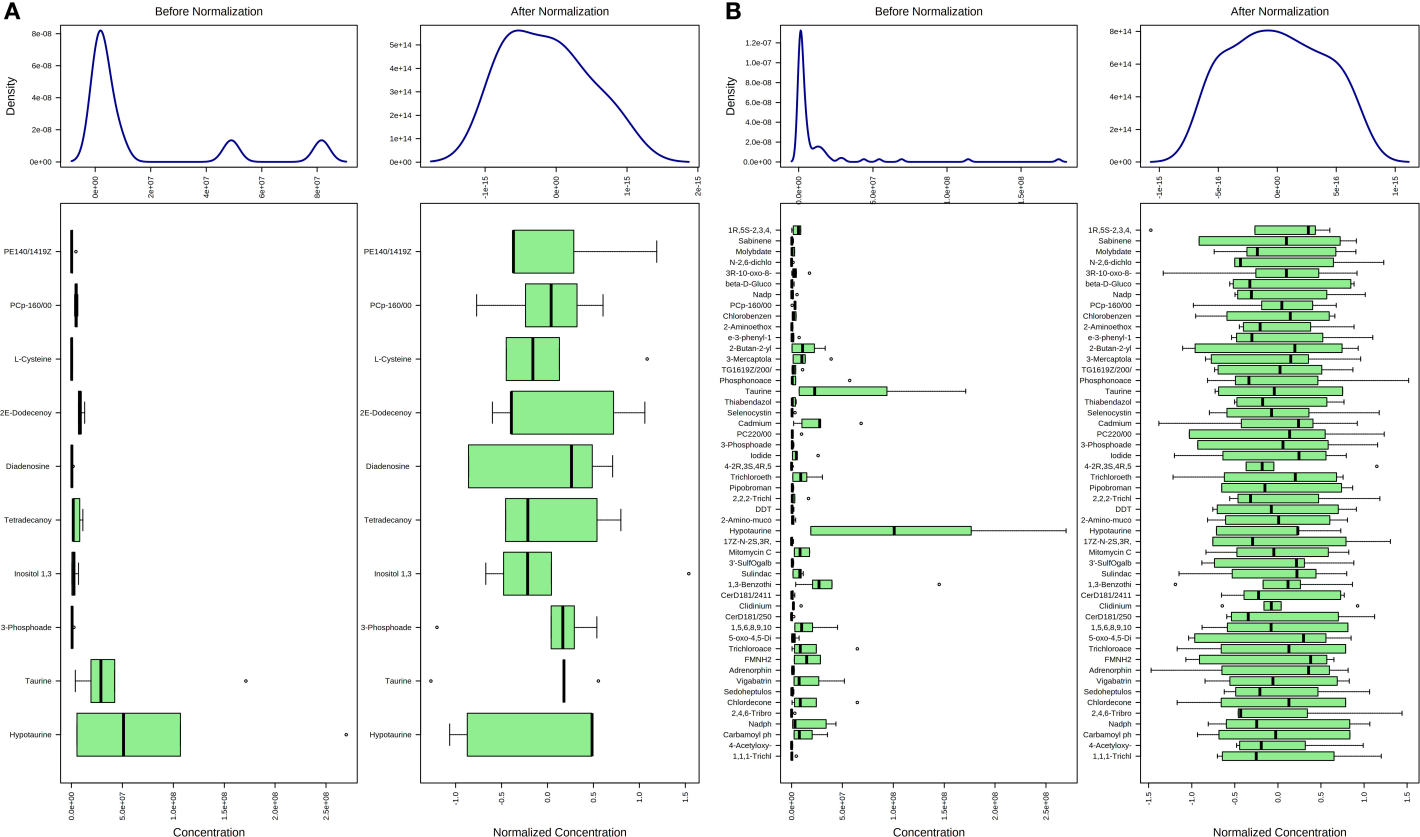
Normalization of common metabolites of spermatozoa **(A)** and seminal plasma **(B)** of both groups, prior to statistical analysis based on quantile normalization, log transformation and pareto scaling.

**Figure 3:**
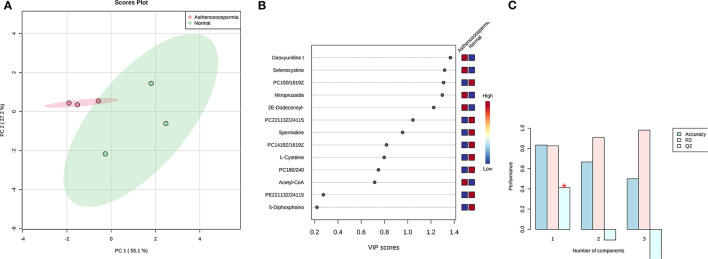
PLS-DA based on score plot **(A)** and Heat map **(B)** of Vip score of significant metabolites in spermatozoa of astheno-oligozoospermia bulls. **(C)** Cross validation of PLS-DA based on score plot of significant metabolites in spermatozoa of astheno-oligozoospermia bulls where Q2 is an estimate of the predictive ability of the model, and is calculated via cross validation. In each CV, the predicted data are compared with the original data, and the sum of squared error is calculated. The prediction error is summed over all samples (Predicted residual sum of squares or PRESS). For convivence, the PRESS is divided by initial sum of the squares and subtracted from 1 to resemble the scale of the R2. Good prediction will have low PRESS or high Q2. It is also possible to have negative Q2, which means that the model is not at all predictive or overfitted.

**Figure 4:**
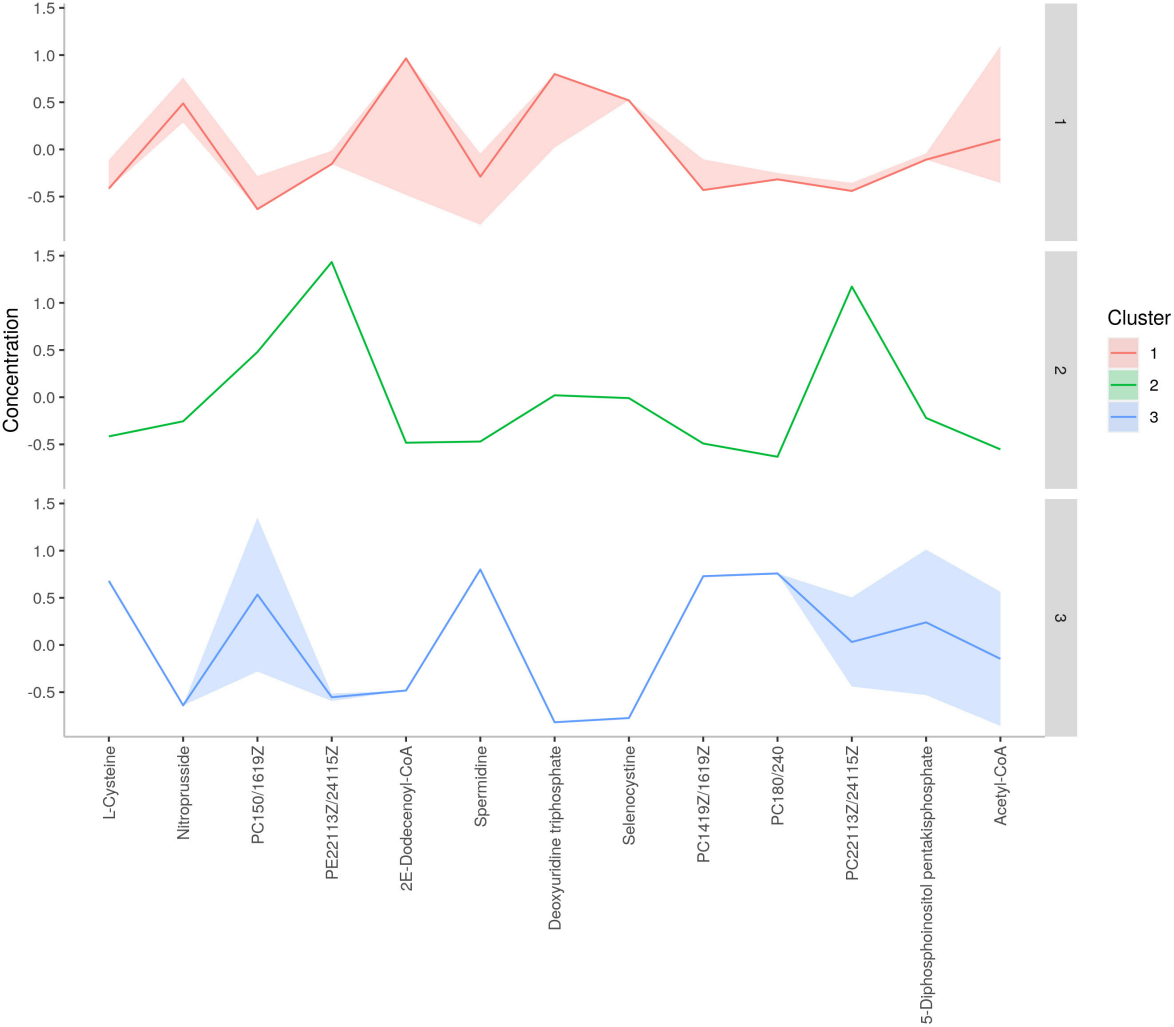
*K*-means clustering of spermatozoa metabolites of astheno-oligozoospermia bulls.

Concentration data of common metabolites between the two groups were normalized to quantile normalization, log transformation, and pareto-scaled (Figures 2A,B). A total of 62 potentially significant features were found to be differentially expressed in the spermatozoa of astheno-oligozoozpermic bulls as compared with normozoospermic bulls (Supplementary Table 1 and Supplementary Figure 3A). Among the dysregulated metabolites, the top 15 metabolites were selected based on pathway enrichment analysis; univariate fold change analysis of these metabolites with threshold 2 identified 12 important features in spermatozoa of asthenozoo-oligozoospermic bulls, of which seven were upregulated, and five were downregulated (Supplementary Figure 3B). Potentially significant (*p* < 0.05) differentially expressed features were shortlisted based on a volcano plot in MetaboAnalyst, which is a combination of *t*-test and fold change. Selenocysteine, deoxyuridine triphosphate, and nitroprusside (Supplementary Table 2 and Supplementary Figure 4) were found to be significantly upregulated in the spermatozoa of astheno-oligozoozpermic bulls. Additionally, PLSDA (multivariate analysis, two-dimensional score plot of PLSDA) and K-means of these metabolites indicated a clear clustering between the groups (Figures 3A–C, 4). Graphical representation of abundance ratio of metabolites above 1 VIP score (Supplementary Figures 5A–C) also highlighted the differences at metabolome level between spermatozoa of both the groups. Metabolites with VIP score (variable importance in projection) ≥ 1 were deoxyuridine triphosphate, selenocysteine, phosphatidylcholine, nitroprusside, and 2E-dodecenoyl-CoA. The relative concentration of corresponding metabolites based on VIP score also revealed higher levels of selenocysteine, deoxyuridine triphosphate, and nitroprusside in spermatozoa of astheno-oligozoospermic bulls as compared with normozoospermic bulls. On the other hand, principal component analysis (Supplementary Figure 6A) revealed the variance of significant features, whereas correlation plot analysis showed the positive correlation of selenocysteine with nitroprusside and deoxyuridine triphosphate (Supplementary Figure 6B).

**Figure 5:**
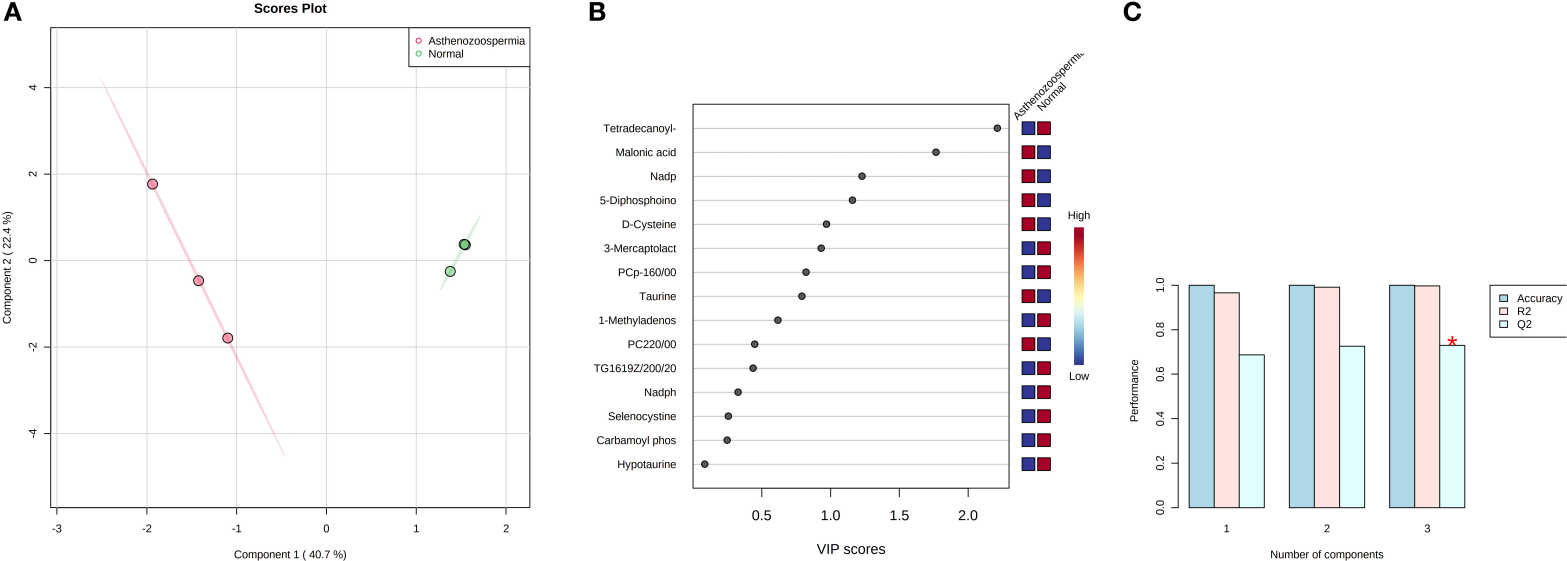
PLS-DA based on score plot **(A)** and Heat map **(B)** of Vip score of significant metabolites in seminal plasma of astheno-oligozoospermia bulls. **(C)** Cross validation of PLS-DA based on score plot of significant metabolites in seminal plasma of astheno-oligozoospermia bulls where Q2 is an estimate of the predictive ability of the model, and is calculated via cross validation. In each CV, the predicted data are compared with the original data, and the sum of squared error is calculated. The prediction error is summed over all samples (Predicted residual sum of squares or PRESS). For convivence, the PRESS is divided by initial sum of the squares and subtracted from 1 to resemble the scale of the R2. Good prediction will have low PRESS or high Q2. It is also possible to have negative Q2, which means that the model is not at all predictive or overfitted.

**Figure 6:**
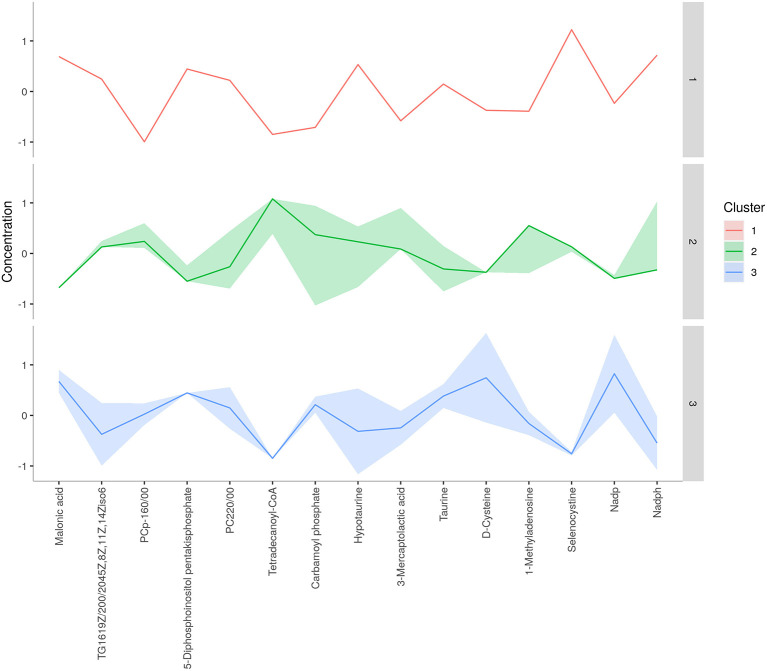
*K*-means clustering of seminal plasma metabolites of astheno-oligozoospermia bulls.

A total of 56 potentially significant dysregulated features were extracted in the seminal plasma (Supplementary Table 3 and Supplementary Figure 7A) of astheno-oligozoozpermic bulls as compared with normozoospermic bulls. Based on pathway enrichment analysis, 12 dysregulated metabolites were shortlisted. Univariate fold change analysis of these metabolites with threshold 2 revealed eight (three metabolites upregulated and five metabolites downregulated) important features in seminal plasma of asthenozoospermic as compared with normozoospermic bulls (Supplementary Figure 7B). In the *t*-test (*p* < 0.05), only three significant features (malonic acid, 5-diphosphoinositol pentakisphosphate, and tetradecanoyl-CoA) were observed (Supplementary Figure 8A). However, volcano plot analysis also revealed that malonic acid and 5-diphosphoinositol pentakisphosphate were significantly upregulated, whereas tetradecanoyl-CoA was significantly downregulated in the seminal plasma of astheno-oligozoospermic bulls (Supplementary Table 4 and Supplementary Figure 8B). Multivariate analysis, two-dimensional score plots of PLSDA (Figures 5A–C) and K-means (Figure 6) indicated a clear clustering between the groups. Metabolites with a VIP score of more than 1.5 included tetradecanoyl-CoA and malonic acid, whereas a VIP score of more than 1 was nicotinamide adenine dinucleotide phosphate (NADP), 5-diphosphoinositol pentakisphosphate, and D-cysteine (Supplementary Figures 9A–E). The principal component analysis also showed the variance of filtered data (Supplementary Figure 10A). Among these compounds, tetradecanoyl-CoA had the highest VIP score, followed by malonic acid, NADP, 5-diphosphoinositol pentakisphosphate, and D-cysteine. The colored boxes of the relative concentration of metabolites, based on VIP score, also showed a low concentration of tetradecanoyl-CoA whereas a high concentration of NADP, D-cysteine, malonic acid, and 5-diphosphoinositol pentakisphosphate in seminal plasma of astheno-oligozoospermic as compared with normozoospermic bulls. Based on univariate analysis, it was found that malonic acid was inversely associated with tetradecanoyl-CoA whereas positively correlated with NADP and 5-diphosphoinositol pentakisphosphate (Supplementary Figure 10B).

### Pathways Enrichment Analysis of Unique Metabolites

The results of an enrichment pathway analysis of unique metabolites in spermatozoa of astheno-oligozoospermic and normozoospermic bulls are presented in Tables 1, 2. The metabolome view of the graphical output of pathway analysis is presented in Supplementary Figures 11A,B. In spermatozoa of astheno-oligozoospermic bulls, metabolism of fatty acid and fatty acid elongation in mitochondria were the significant (*P*-values from 0.02 to 0.05) pathways, whereas butanoyl-CoA, aceto-acetyl-CoA, tetradecanoyl-CoA, hydroxy octanoyl-CoA, 3-oxooctanoyl- CoA, and 3-oxododecanoyl-CoA were the observed unique metabolites. On the other hand, glycerophospholipid metabolism (*P*-value of 0.03) was the significant pathway observed in spermatozoa of normozoospermic bulls, whereas phosphatidylcholine, phosphatidylethanolamine, and PS (16:0/16:0) were the enriched metabolites.

**Table 1:** Enrichment of pathways involved in unique metabolites of spermatozoa in normozoospermia bulls.

**Pathways**	**Total**	**Expected**	**Hits**	**Raw** ***p***	**Impact**
Fatty acid metabolism	39	3.00	7	0.03	0.23
Fatty acid elongation in mitochondria	27	2.08	5	0.05	0.16
Taurine and hypotaurine metabolism	7	0.54	2	0.10	0.75
Pyruvate metabolism	22	1.69	3	0.24	0.00
Pantothenate and CoA biosynthesis	15	1.15	2	0.32	0.14
Linoleic acid metabolism	5	0.38	1	0.33	0.00
Synthesis and degradation of ketone bodies	5	0.38	1	0.33	0.13
Inositol phosphate metabolism	28	2.15	3	0.37	0.06
Thiamine metabolism	7	0.54	1	0.43	0.00
Propanoate metabolism	20	1.54	2	0.46	0.00
Butanoate metabolism	20	1.54	2	0.46	0.33
Alpha-linolenic acid metabolism	9	0.69	1	0.51	0.00
Nitrogen metabolism	9	0.69	1	0.51	0.00
Ascorbate and aldarate metabolism	9	0.69	1	0.51	0.40
Nicotinate and nicotinamide metabolism	13	1.00	1	0.65	0.24
Glycerophospholipid metabolism	29	2.23	2	0.67	0.24
Glycosylphosphatidylinositol (GPI)-anchor biosynthesis	14	1.08	1	0.68	0.02
Selenoamino acid metabolism	15	1.15	1	0.70	0.00
Terpenoid backbone biosynthesis	15	1.15	1	0.70	0.00
Pentose and glucuronate interconversions	15	1.15	1	0.70	0.33
Glyoxylate and dicarboxylate metabolism	16	1.23	1	0.72	0.00
Folate biosynthesis	16	1.23	1	0.72	0.21
beta-Alanine metabolism	17	1.31	1	0.75	0.00
Fructose and mannose metabolism	19	1.46	1	0.78	0.02
Amino sugar and nucleotide sugar metabolism	37	2.85	2	0.79	0.03
Pyrimidine metabolism	37	2.85	2	0.79	0.08
Citrate cycle (TCA cycle)	20	1.54	1	0.80	0.05
Lysine degradation	20	1.54	1	0.80	0.09
Valine leucine and isoleucine degradation	38	2.92	2	0.81	0.08
Sphingolipid metabolism	21	1.62	1	0.82	0.00
Tryptophan metabolism	41	3.15	2	0.84	0.06
Alanine aspartate and glutamate metabolism	23	1.77	1	0.84	0.00
Starch and sucrose metabolism	23	1.77	1	0.84	0.11
Tyrosine metabolism	42	3.23	2	0.85	0.07
Arginine and proline metabolism	44	3.38	2	0.87	0.02
Cysteine and methionine metabolism	28	2.15	1	0.90	0.00
Drug metabolism—other enzymes	30	2.31	1	0.91	0.00
Arachidonic acid metabolism	36	2.77	1	0.95	0.00
Fatty acid biosynthesis	38	2.92	1	0.95	0.06
Biosynthesis of unsaturated fatty acids	42	3.23	1	0.97	0.00
Purine metabolism	68	5.23	2	0.97	0.01
Primary bile acid biosynthesis	46	3.54	1	0.98	0.03

**Table 2:** Enrichment of pathways involved in unique metabolites of spermatozoa in normozoospermia bulls.

**Pathways**	**Total**	**Expected**	**Hits**	**Raw** ***p***	**Impact**
Glycerophospholipid metabolism	29	0.77	3	0.04	0.27
Linoleic acid metabolism	5	0.13	1	0.13	0.00
Alpha-linolenic acid metabolism	9	0.24	1	0.22	0.00
Pyrimidine metabolism	37	0.98	2	0.26	0.10
Caffeine metabolism	12	0.32	1	0.28	0.00
Glycosylphosphatidylinositol (GPI)-anchor biosynthesis	14	0.37	1	0.32	0.02
Glyoxylate and dicarboxylate metabolism	16	0.43	1	0.35	0.00
Folate biosynthesis	16	0.43	1	0.35	0.00
Citrate cycle (TCA cycle)	20	0.53	1	0.42	0.05
Sphingolipid metabolism	21	0.56	1	0.43	0.28
Pyruvate metabolism	22	0.59	1	0.45	0.00
Alanine, aspartate, and glutamate metabolism	23	0.61	1	0.46	0.00
Porphyrin and chlorophyll metabolism	25	0.66	1	0.49	0.06
Glutathione metabolism	26	0.69	1	0.51	0.00
Arachidonic acid metabolism	36	0.96	1	0.63	0.00
Tyrosine metabolism	42	1.12	1	0.68	0.00

The results of enrichment of pathways of unique metabolites in seminal plasma of astheno-oligozoospermic and normozoospermic bulls are presented in Tables 3, 4, and the metabolome view of the graphical output of the pathway analysis are presented in Supplementary Figures 12A,B. Synthesis and degradation of ketone bodies, pyruvate metabolism, and inositol phosphate metabolism (*P*-values from 0.006 to 0.03) were the significant metabolic pathways enriched in the seminal plasma of astheno-oligozoospermic bulls. Frequently observed metabolites in these significant pathways were acetyl-CoA and aceto-acetyl-CoA, L-malic acid, myo-inositol hexakisphosphate, and inositol 1,3,4-trisphosphate. In contrast, no significant pathways were found in the seminal plasma of normozoospermia bulls (*P*-values from 0.4 to 1.17).

**Table 3:** Enrichment of pathways involved in unique metabolites of seminal plasma in astheno-oligozoospermia bulls.

**Pathway name**	**Total**	**Expected**	**Hits**	**Raw** ***p***	**Impact**
Synthesis and degradation of ketone bodies	5	0.13	2	0.01	0.13
Pyruvate metabolism	22	0.59	3	0.02	0.14
Inositol phosphate metabolism	28	0.74	3	0.04	0.06
Pantothenate and CoA biosynthesis	15	0.40	2	0.06	0.14
Terpenoid backbone biosynthesis	15	0.40	2	0.06	0.00
Glyoxylate and dicarboxylate metabolism	16	0.43	2	0.07	0.00
Valine leucine and isoleucine degradation	38	1.01	3	0.08	0.06
Fatty acid metabolism	39	1.04	3	0.08	0.31
Butanoate metabolism	20	0.53	2	0.10	0.23
Lysine degradation	20	0.53	2	0.10	0.09
Citrate cycle (TCA cycle)	20	0.53	2	0.10	0.06
Propanoate metabolism	20	0.53	2	0.10	0.01
Linoleic acid metabolism	5	0.13	1	0.13	0.00
Ascorbate and aldarate metabolism	9	0.24	1	0.22	0.40
Alpha-linolenic acid metabolism	9	0.24	1	0.22	0.00
Tryptophan metabolism	41	1.09	2	0.30	0.00
Pentose and glucuronate interconversions	15	0.40	1	0.33	0.33
Folate biosynthesis	16	0.43	1	0.35	0.21
Beta-alanine metabolism	17	0.45	1	0.37	0.00
Starch and sucrose metabolism	23	0.61	1	0.46	0.11
Glycolysis or Gluconeogenesis	26	0.69	1	0.51	0.04
Fatty acid elongation in mitochondria	27	0.72	1	0.52	0.25
Cysteine and methionine metabolism	28	0.74	1	0.53	0.00
Glycerophospholipid metabolism	29	0.77	1	0.55	0.15
Steroid biosynthesis	35	0.93	1	0.62	0.00
Arachidonic acid metabolism	36	0.96	1	0.63	0.00
Pyrimidine metabolism	37	0.98	1	0.64	0.04
Amino sugar and nucleotide sugar metabolism	37	0.98	1	0.64	0.02
Fatty acid biosynthesis	38	1.01	1	0.65	0.03
Tyrosine metabolism	42	1.12	1	0.68	0.00
Purine metabolism	68	1.81	1	0.85	0.00

**Table 4:** Enrichment of pathways involved in unique metabolites of seminal plasma in normozoospermia bulls.

**Pathways name**	**Total**	**Expected**	**Hits**	**Raw** ***p***	**Impact**
Fatty acid elongation in mitochondria	27	0.47	2	0.08	0.07
Glycerophospholipid metabolism	29	0.50	2	0.09	0.24
Pyrimidine metabolism	37	0.64	2	0.13	0.12
Fatty acid metabolism	39	0.67	2	0.14	0.04
Drug metabolism—cytochrome P450	56	0.97	2	0.25	0.06
Linoleic acid metabolism	5	0.09	1	0.08	0.00
Alpha-linolenic acid metabolism	9	0.16	1	0.15	0.00
Glycosylphosphatidylinositol (GPI)-anchor biosynthesis	14	0.24	1	0.22	0.02
Inositol phosphate metabolism	28	0.48	1	0.39	0.06
Arachidonic acid metabolism	36	0.62	1	0.47	0.00
Valineleucine and isoleucine degradation	38	0.66	1	0.49	0.08
Purine metabolism	68	1.17	1	0.70	0.00

### Visualization of Metabolic Network

**Figure 7:**
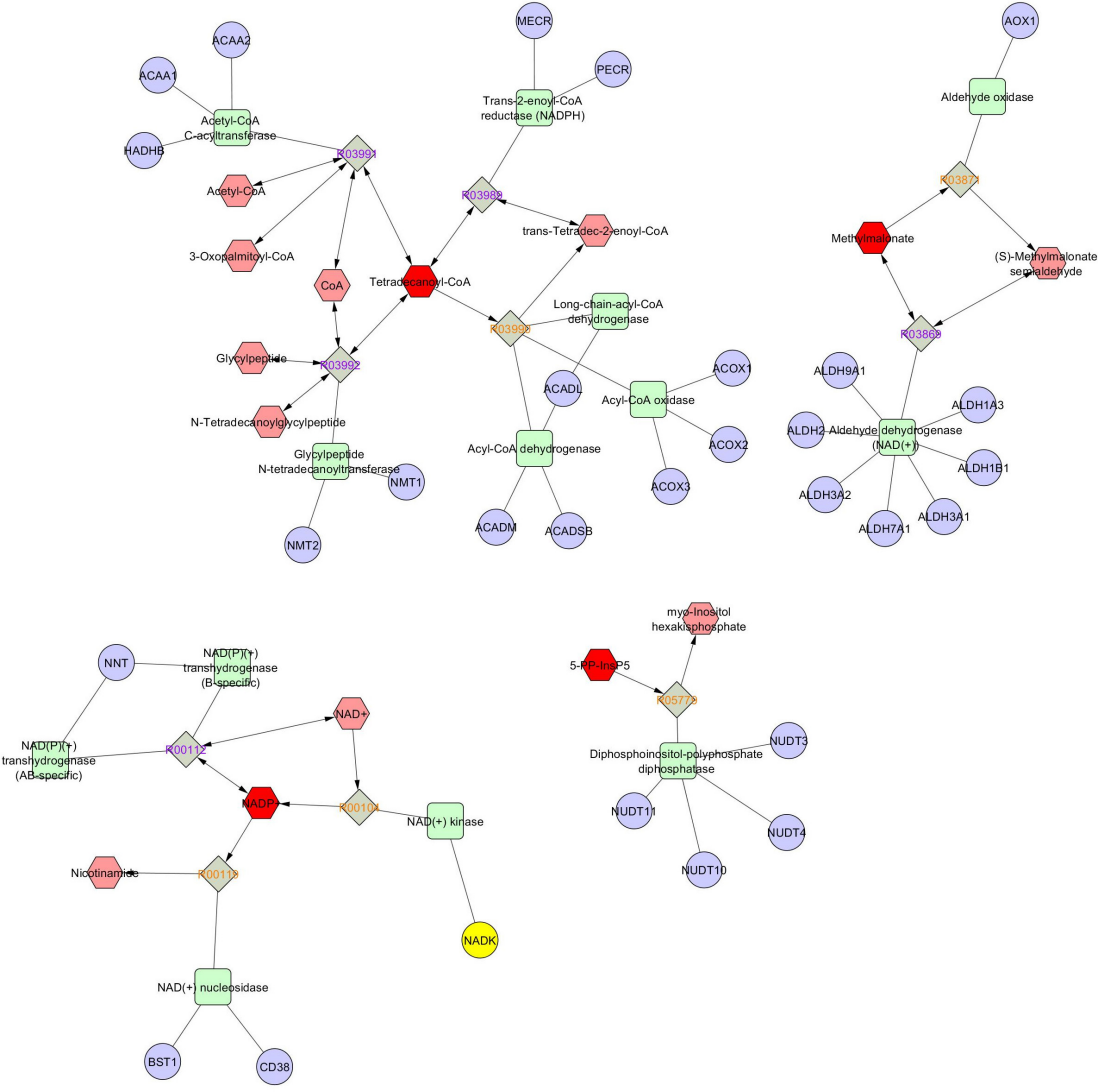
Metscape of network based on compound-enzyme-gene reaction.

Metabolic network based on compound–enzyme–gene reaction of most abundant metabolites in seminal plasma such as tetradecanoyl-CoA, NADP, 5-diphosphoinositol pentakisphosphate, and malonic acid (VIP score > 1, Figure 7) was illustrated using MetScape database (a plugin for Cytoscape). NADP+ undergoes a reversible reaction to produce sorbitol and is finally converted to D-fructose. Tetradecanoyl-CoA undergoes reversible reactions to produce acetyl-CoA and 3-oxopalmitoyl-CoA, whereas methylmalonate reversibly and irreversibly produces s-methylmalonate semialdehyde, and 5-diphosphoinositol pentakisphosphate irreversibly produces myo-inositol hexakisphosphate, which plays an obligatory role to produce energy to support sperm motility. However, a metabolic network for selenocysteine, deoxyuridine triphosphate, and nitroprusside was not found in the MetScape database.

## Discussion

Poor semen quality in breeding bulls jeopardizes the genetic improvement through artificial breeding. Although breeding bulls are selected based on qualifying all the breeding soundness evaluation tests, a significant proportion of bulls, especially the crossbred bulls, produce inferior semen quality. Asthenozoospermia and oligozoospermia are the two conditions commonly found in infertile/subfertile bulls; however, the etiology remains poorly understood. A plethora of researchers has identified discriminatory metabolites in sperm and seminal plasma such as selenocysteine, hypotaurine, D-cysteine, L-malic acid, phosphatidylcholine, and spermidine in low-fertile and high-fertile bulls (4), non-obstructive azoospermia (17), asthenozoospermia (27), oligozoospermia (22), and asthenozoospermia in men (28). However, such information is very limited in the case of bulls. In the current study, using the non-targeted metabolomics technique, we performed the metabolic characterization of spermatozoa and seminal plasma from astheno-oligozoospermic bulls. For the first time, we report here the metabolomic alterations in spermatozoa and seminal plasma of astheno-oligozoospermic bulls as compared with normozoospermic bulls.

We observed a significantly higher abundance ratio of selenocysteine, deoxyuridine triphosphate, and nitroprusside in spermatozoa of astheno-oligozoospermic bulls as compared with normozoospermic bulls. Selenocysteine, an analog of selenium, is reported to have a positive correlation with spermatogenesis and male fertility in rats and ram (29). It is well-documented that both deficiency and excess of selenium in the diet had an adverse effect on the seminiferous epithelium, spermatogenesis, and maturation process of spermatozoa in the epididymis, sperm motility, morphology, and bull fertility (30, 31). In mice, it is reported that excess selenium was associated with the frequent presence of equidistant, cross-sectioned midpieces of the tail embedded in a common cytoplasm, which is indicative of loss of sperm motility (32). Deoxyuridine triphosphate has a strong affinity for damaged DNA, and mis-inferences can bring adverse effects to sperm DNA, male fertility, and reproductive outcomes (33). On the other hand, exogenous deoxyuridine triphosphate-conjugated fluorescein isothiocyanate is extensively used to measure sperm DNA fragmentation (%) due to its strong affinity for damaged DNA (HMDB). There are plenty of reports showing a significantly higher level of DNA damage in astheno- oligozoospermia (34, 35). Recently, a comparative study of metabolomic profile between crossbred bull spermatozoa and zebu bull spermatozoa revealed a significantly higher level of deoxyuridine triphosphate in spermatozoa of crossbreed bulls (Mohua et al., unpublished data). We observed a significantly higher level of nitroprusside in a spermatozoal extract of astheno-oligozoospermic bulls. Nitroprusside, a nitric oxide donor, has been shown to have a biphasic role in spermatozoa regulating sperm capacitation, acrosome reaction, and male fertility (36). On the one hand, it was shown that sodium nitroprusside had a positive effect on spermatozoa to counterbalance the increased level of ROS and decreased level of adenosine triphosphate (ATP)/Ca^2+^, whereas, on the other hand, Balercia et al. (37) and Bolaños et al. (38) reported a negative effect on asthenozoospermic men. Similarly, Khodaei et al. (39) observed both lucrative and malefic effects of exogenous supplementation of sodium nitroprusside on a frozen-thawed bull and buffalo sperm morphology, motility, and viability. A concentration-dependent effect of sodium nitroprusside with deleterious effect on sperm motility at higher concentrations and beneficial effect on sperm functions at lower concentrations have been reported earlier (40).

Another intriguing finding is the decreased level of tetradecanoyl-CoA in seminal plasma of astheno-oligozoospermia bulls as compared with normozoospermia bulls. Tetradecanoyl-CoA, also known as myristoyl-CoA, is involved in myristoylation, which is a posttranslational modification in spermatozoa that regulates the glycolytic pathway required for flagellar activity. During the epididymal maturation process, myristoylated protein plays a paramount role in sperm membrane lipid distribution, activating the receptors and triggering the signaling cascades (41). A myristoylated protein of seminal plasma in the efflux of cholesterol and phospholipid in epididymal sperm and thus confirming a role in capacitation, acrosome reaction, and fertilization process. Our results, coupled with the earlier observations, indicate that a lower concentration of tetradecanoyl-CoA may be responsible for the alteration of the myristoylation mechanism and flagellar activity in astheno-oligozoospermic bulls. Cysteine was significantly higher in the seminal plasma of astheno-oligozoospermic bulls as compared with normozoospermic bulls. Cysteine is a non-essential amino acid with antioxidative properties and is mainly known as a scavenger of ROS. Basically, the generation of cysteine depends on the sulfur-containing amino acids diet, and the breakdown of cysteine by oxidative pathways leads to more production of taurine (42), which has an additive effect on sperm motility. In the same context, Zhang et al. (19) reported a higher level of cysteine and taurine in the seminal plasma of asthenozoospermia patients and manifested a cardinal role in antioxidative stress cascade. In the current study, heat map analysis of VIP score plot also revealed elevated level cysteine and taurine in seminal plasma of astheno-oligozoospermic bulls as compared with a seminal plasma of normozoospermic bulls, indicating the possibilities of a significant rise in antioxidative defense mechanism to nullify the effect of ROS, which causes extensive damage to the sperm motility and male fertility.

It is important to note that seminal plasma of astheno-oligozoospermia bulls showed a higher level of malonic acid as compared with a seminal plasma of normozoospermia bulls, which act as a competitive inhibitor of succinic dehydrogenase and are responsible for dehydrogenation of succinate in the Krebs cycle (43). Malonic acid has a spermicidal effect; even an addition of 0.1% rendered the sperm immotile (44). Inhibition of any of the intermediate substrates in the Krebs cycle delimits the utilization of carbohydrates as energy in the sperm. An old pioneering study also reported that malonate inhibits respiration and motility of ejaculated spermatozoa in the absence of glucose (45). Thus, it could be possible that a higher level of malonic acid in the seminal plasma of astheno-oligozoospermic bulls might have impaired sperm motility.

5-Diphosphoinositol pentakisphosphate, which is irreversibly converted into myo-inositol hexakisphosphate, was found to be significantly higher in seminal plasma of astheno-oligozoospermic bulls. Myo-inositol plays an important role in the maturation and migration of male gametes in the epididymis (46) and ameliorates the motility of sperm in oligoasthenoteratozoospermia men (47). Our findings that astheno-oligozoospermic bulls had higher concentrations of 5-diphosphoinositol pentakisphosphate coupled with myo-inositol hexakisphosphate and IP_3_ indicate the possibilities of elevated Ca^2+^-dependent intracellular signal transductions and protein phosphorylation to produce more ATP to counterbalance the motility of partially motile/immotile sperm in astheno-oligozoospermic bulls as compared with normozoospermic bulls. We identified a significantly higher level of NADP, a coenzyme present at lower concentration and functions apace with many enzymes of glycolysis and tricarboxylic acid (TCA) cycle in sperm to conserve energy (48), in astheno-oligozoospermic bulls. Presumably, upregulation of NADP in seminal plasma astheno-oligozoospermic bulls might be a mechanism to conserve more energy to maintain the altered physiological functions of flagellar movement.

Among the several altered pathways, fatty acid metabolism and fatty acid elongation in mitochondria were significantly observed in spermatozoa of astheno-oligozoospermic bulls. Basically, the fatty acid is an integral component of triglycerides and ketone bodies, and phospholipids take an active part in the generation of energy and formation of the lipid bilayer of sperm. Generally, long-chain fatty acid increases the stringency of the sperm plasma membrane and baffles the fertilization events. In this context, Tang et al. (49) reported a higher level of saturated and unsaturated fatty acids in the seminal plasma of asthenozoospermic men. Therefore, significantly higher levels of fatty acid metabolism and fatty acid elongation in mitochondria in astheno-oligozoospermic bulls might be associated with higher production of energy, altered phospholipid metabolism, and possibly instigated poor sperm motility.

On the other hand, synthesis and degradation of ketone bodies, pyruvate metabolism, and inositol phosphate metabolism were significantly observed in the seminal plasma of astheno-oligozoospermia bulls, and top metabolites observed include acetyl-CoA and acetoacetyl-CoA, indicating rapid degradation of ketone bodies into acetyl-CoA. Generally, ketone bodies play an important role in sperm motility by providing energy in the form of ATP (50). It is well-illustrated that sperm motility relies on ATP, and glycolysis and oxidative phosphorylation are the two potent ways to generate ATP in sperm cells (51). Basically, the generation of ATP takes place specifically in mitochondria, localized in the midpiece of the sperm tail, where the TCA cycle is the predominant metabolic pathway. Acetyl-CoA released from synthesis and degradation of ketone bodies enters the TCA cycle for the generation of energy in the form of ATP. Acetoacetyl-CoA is cleaved by the thiolase enzyme into two molecules of acetyl-CoA, which further enter into the Kerbs cycle for the generation of energy (52). Several researchers have found a positive correlation between sperm motility and concentration with ATP production (19, 53). Besides, L-malic acid, myo-inositol hexakisphosphate, and inositol 1,3,4-trisphosphate also take part in energy production for sperm motility and male fertility. Earlier reports by Zhang et al. (54) in oligozoospermia, Jayaraman et al. (55) in azoospermia, and Zhang et al. (19) in asthenozoospermia showed alteration in energy consumption and antioxidant defense mechanism. Our results coupled with earlier findings indicate an altered TCA cycle in the semen of astheno-oligozoospermia bull; in other words, energy production was accelerated to meet the energy demand, but unfortunately, the smaller number of motile spermatozoa present in astheno-oligospermic bulls failed to utilize the available energy resulting in increased concentrations of energy metabolism-related metabolites in seminal plasma.

In contrast, glycerophospholipid metabolism was found to be the topmost pathway in spermatozoa, and seminal plasma of normozoospermic bulls and the metabolites observed include phosphatidylcholine, phosphatidylethanolamine, and PS (16:0/16:0). These molecules are the predominant constituents of the sperm cell membrane and seminal plasma. Plasma membrane lipidic composition such as phospholipid, glycerophospholipid, and cholesterol regulates the membrane fluidity, sperm motility, capacitation, acrosome reaction, and a complex cascade of fertilization. These lipid bilayer membranes undergo marked changes during spermatogenesis and epididymal maturation (56, 57). However, these significant changes are probably due to the efflux of cholesterol and phospholipid between sperm and seminal plasma (58). A plethora of researchers have recorded a higher level of phospholipid in seminal plasma of azoo-oligospermia patients and showed a highly significant correlation of metabolites between seminal plasma and sperm membrane lipids (56, 57). In the same context, Lucio et al. (57) marked phosphatidylcholine and phosphatidylethanolamine as lipid markers of sperm motility in normozoospermic dogs, whereas no lipid markers were found in asthenozoospermic dog semen samples. Similarly, we found phosphatidylcholine and phosphatidylethanolamine exclusively in spermatozoa and seminal plasma of normozoospermia bulls suggesting that the altered level or drastic loss of phosphatidylcholine, phosphatidylethanolamine, and PS (16:0/16:0) from sperm and seminal plasma may be one of the predominant reasons for poor sperm motility and idiopathic infertility in bulls. Although the sample size could be a limitation of the present study, the sensitivity of the LC-MS/MS approach and the use of true representative samples under each category, along with strong bioinformatic analysis, enabled us to generate detailed metabolomic differences between astheno-oligozoospermic and normospermic bulls. Thus, we believe that the sample size used in the study is appropriate to derive the conclusions.

## Conclusion

Collectively, for the first time using LC-MS/MS-based approach, the present study revealed vital information related to semen metabolomic differences between astheno-oligozoospermic and normospermic crossbred breeding bulls. In astheno-oligozoospermic bulls, selenocysteine, deoxyuridine triphosphate, and nitroprusside were significantly abundant in spermatozoa, whereas malonic acid, 5-diphosphoinositol pentakisphosphate, D-cysteine, and NADP were significantly abundant in seminal plasma. Significantly, the metabolites involved in fatty acid metabolism and fatty acid elongation in mitochondria and synthesis and degradation of ketone bodies were altered in both spermatozoa and seminal plasma of astheno-oligozoospermic bulls. The knowledge generated in the present investigation advances our understanding of astheno-oligozoospermia and can be applied for the modulation of pathways to restore the sperm motility and concentration in astheno-oligozoospermic bulls.

## Data Availability Statement

The datasets presented in this study can be found in online repositories. The names of the repository/repositories and accession number(s) can be found at: https://www.ebi.ac.uk/metabolights/MTBLS1642.

## Ethics Statement

The animal study was reviewed and approved by Animal Ethics Committee of ICAR—National Dairy Research Institute (CPCSEA/IAEC/LA/SRS-ICAR-NDRI-2017/No.09).

## Author Contributions

MD carried out her PhD research under the guidance of AK. AK, TKD, and MD designed the experiment. MD, AK, and TKD wrote and reviewed and edited the manuscript. MKS, PN, MAMK, MD, KKS, GK, TSK, and PKM carried out the formal analysis of metabolites. KR, AM, and SJ helped in statistical analysis. All authors contributed to the article and approved the submitted version.

## Funding

This work was supported by the Bill and Melinda Gates Foundation (grant number OPP1154401) under the project Molecular markers for improving reproduction in cattle and buffaloes.

## Conflict of Interest

The authors declare that the research was conducted in the absence of any commercial or financial relationships that could be construed as a potential conflict of interest.

## Publisher's Note

All claims expressed in this article are solely those of the authors and do not necessarily represent those of their affiliated organizations, or those of the publisher, the editors and the reviewers. Any product that may be evaluated in this article, or claim that may be made by its manufacturer, is not guaranteed or endorsed by the publisher.
