# Supplementary material for: Deep Metabolomic Profiling Reveals Alterations in Fatty Acid Synthesis and Ketone Body Degradations in Spermatozoa and Seminal Plasma of Astheno-Oligozoospermic Bulls
Source: Front Vet Sci. 2022 Jan 11;8:755560. doi: 10.3389/fvets.2021.755560 (PMC8787163; doi:10.3389/fvets.2021.755560)
Supplement: Supplementary file 1 [file Data_Sheet_1.docx]

**Supplementary files (Tables)**

**Manuscript Title:** Deep metabolomic profiling reveals alterations in fatty acid synthesis and ketone body degradations in spermatozoa and seminal plasma of astheno-oligozoospermic bulls

**Authors:** Mohua Dasgupta, Arumugam Kumaresan^*^, Kaustubh Kishor Saraf, Pradeep Nag, Manish Kumar Sinha, Muhammad Aslam M. K, Gayathree Karthikkeyan, T. S. Keshava Prasad, Prashant Kumar Modi, Tirtha Kumar Datta, Kerekoppa Ramesha, Ayyasamy Manimaran, Sakthivel Jeyakumar

**Supplementary Table 1:** Differentially expressed metabolites identified by fold change analysis with threshold2

| Metabolites in spermatozoa | Expression ratio | log2(FC) |
| --- | --- | --- |
| Rofecoxib | 0.005741 | -7.4446 |
| Lactyl-CoA | 0.008273 | -6.9173 |
| Galabiosylceramide D181/220 | 0.010742 | -6.5406 |
| S-5-Diphosphomevalonic acid | 0.014225 | -6.1355 |
| 2-Aminoethoxy2R-3-docosanoyloxy-2-1Z,11Z-octadeca-1,11-dien-1-yloxypropoxyphosphinic acid | 0.017771 | -5.8143 |
| Adenosine 5'-hexahydrogen pentaphosphate | 0.018196 | -5.7803 |
| 2-Aminoethoxy2R-2-1Z-hexadec-1-en-1-yloxy-3-pentadecanoyloxypropoxyphosphinic acid | 0.018789 | -5.734 |
| PC1419Z/1619Z | 0.034881 | -4.8414 |
| PC180/240 | 0.044009 | -4.506 |
| Levomethadyl acetate | 0.049372 | -4.3402 |
| PC150/1619Z | 0.051019 | -4.2928 |
| Benzthiazide | 0.051349 | -4.2835 |
| PE22113Z/24115Z | 0.054003 | -4.2108 |
| 3,5-Diiodo-4-hydroxyphenylpyruvic acid | 0.059875 | -4.0619 |
| CerD181/260 | 0.062382 | -4.0027 |
| Thallium monochloride | 0.06286 | -3.9917 |
| L-Cysteine | 0.070789 | -3.8203 |
| Amantadine | 0.075192 | -3.7333 |
| Spermidine | 0.085737 | -3.5439 |
| Flecainide | 0.10043 | -3.3157 |
| 5-Diphosphoinositol pentakisphosphate | 0.13656 | -2.8724 |
| TG1619Z/200/2045Z,8Z,11Z,14Ziso6 | 0.14298 | -2.8061 |
| Acrylyl-CoA | 0.14487 | -2.7872 |
| 2-hydroxy2S,3R-3-hydroxy-2-1-hydroxyhexadecylideneaminooctadecyloxyphosphoryloxyethyltrimethylazanium | 0.15068 | -2.7304 |
| Trichloroacetic acid | 0.27006 | -1.8887 |
| Lithocholyltaurine | 0.33436 | -1.5805 |
| 3-Nitropropanoic acid | 0.48017 | -1.0584 |
| Tricosane | 2.0519 | 1.0369 |
| 2-3-docosanoyloxy-2-1Z,11Z-octadeca-1,11-dien-1-yloxypropyl phosphonatooxyethyltrimethylazanium | 2.2837 | 1.1913 |
| 2-Aminoethoxy2R-3-7Z,10Z,13Z,16Z-docosa-7,10,13,16-tetraenoyloxy-2-1Z-octadec-1-en-1-yloxypropoxyphosphinic acid | 2.3214 | 1.215 |
| Phosphoadenosine phosphosulfate | 2.362 | 1.24 |
| Phosphoribosyl-ATP | 2.4714 | 1.3053 |
| Tungsten4 | 2.608 | 1.3829 |
| Acetyl-CoA | 2.792 | 1.4813 |
| Copperii sulfate | 2.9791 | 1.5749 |
| Chloroform | 3.0232 | 1.5961 |
| Thiabendazole | 3.1936 | 1.6752 |
| beta-D-Glucosyl-N-dodecanoylsphingosine | 3.4782 | 1.7983 |
| Phytosphingosine | 4.7638 | 2.2521 |
| N-4Z-1-2R,3R,4R,5R,6R-3,4-dihydroxy-6-hydroxymethyl-5-2R,3R,4S,5R,6R-3,4,5-trihydroxy-6-hydroxymethyloxan-2-yloxyoxan-2-yloxy-3-hydroxyoctadec-4-en-2-yltetracosanimidic acid | 5.4547 | 2.4475 |
| Methoxyflurane | 8.049 | 3.0088 |
| DITP | 8.049 | 3.0088 |
| Purine | 9.2385 | 3.2077 |
| Trichloroethene | 16.651 | 4.0576 |
| 2E-Dodecenoyl-CoA | 41.603 | 5.3786 |
| 2-hydroxy2S,3R,4E-3-hydroxy-2-15Z-1-hydroxytetracos-15-en-1-ylideneaminononadec-4-en-1-yloxyphosphoryloxyethyltrimethylazanium | 67.35 | 6.0736 |
| Cadmium | 79.614 | 6.3149 |
| 1,5,6,8,9,10,11,11-Octachloro-4-oxatetracyclo6.2.1.0,.0,undec-9-ene | 84.993 | 6.4093 |
| 3,4,5,21,22,23-Hexahydroxy-8,18-dioxo-12,13-bis3,4,5-trihydroxybenzoyloxy-9,14,17-trioxatetracyclo17.4.0.0,.0,tricosa-123,2,4,6,19,21-hexaen-11-yl 3,4,5-trihydroxybenzoate | 95.673 | 6.58 |
| 2-Aminoethoxy2R-2-1Z-octadec-1-en-1-yloxy-3-pentadecanoyloxypropoxyphosphinic acid | 103.46 | 6.6929 |
| 7-Methylguanosine 5'-diphosphate1 | 108.28 | 6.7586 |
| N-4Z-1-2R,3R,4R,5R,6R-3,4-dihydroxy-6-hydroxymethyl-5-2R,3R,4S,5R,6R-3,4,5-trihydroxy-6-hydroxymethyloxan-2-yloxyoxan-2-yloxy-3-hydroxyoctadec-4-en-2-ylpentacosanimidic acid | 109.12 | 6.7697 |
| Nitroprusside | 111.37 | 6.7992 |
| 4-2R,3S,4R,5R-5-6-amino-9H-purin-9-yl-4-hydroxy-3-phosphonooxyoxolan-2-ylmethoxyhydroxyphosphoryloxyhydroxyphosphoryloxy-2-hydroxy-N-2-2-2-4-hydroxyphenylacetylsulfanylethyl-C-hydroxycarbonimidoylethyl-3,3-dimethylbutanimidic acid | 120.27 | 6.9102 |
| Adenosine tetraphosphate | 123.34 | 6.9465 |
| Deoxyuridine triphosphate | 166.01 | 7.3752 |
| Selenocystine | 175.96 | 7.4591 |
| Guanosine 2',3'-cyclic phosphate | 255.61 | 7.9978 |
| 2,4,6-Tribromophenol | 279.97 | 8.1291 |
| 2-Aminoethoxy2R-2-1Z-hexadec-1-en-1-yloxy-3-5Z,8Z,11Z,14Z,17Z-icosa-5,8,11,14,17-pentaenoyloxypropoxyphosphinic acid | 300.12 | 8.2294 |
| PC22113Z/24115Z | 482.54 | 8.9145 |
| Hexachlorobenzene | 779.3 | 9.606 |

**Supplementary Table 2:** Significant features identified by volcano plot in spermatozoa of astheno-oligzoospermia bulls

| Volcano plot | Retention time | Mass/charge | FC | log2(FC) | p. adjusted at 0.05 |
| --- | --- | --- | --- | --- | --- |
| Selenocystine | 6.65 | 336.96 | 3.9242 | 1.9724 | 0.015128 |
| Deoxyuridine triphosphate | 5.24 | 234.96 | 5.5639 | 2.4761 | 0.047517 |
| Nitroprusside | 23.86 | 216.96 | 4.099 | 2.0353 | 0.005549 |

**Supplementary Table 3:** Differentially expressed metabolites identified by fold change analysis with threshold 2

| Metabolite in seminal plasma | Expression ratio | log2(FC) |
| --- | --- | --- |
| 1,5,6,8,9,10,11,11-Octachloro-4-oxatetracyclo6.2.1.0,.0,undec-9-ene | 0.0021671 | -8.85 |
| Phosphonoacetic acid | 0.0050163 | -7.6392 |
| Tetradecanoyl-CoA | 0.011774 | -6.4082 |
| 4-Bromophenol | 0.017416 | -5.8434 |
| Tiludronic acid | 0.020575 | -5.603 |
| Irbesartan | 0.021709 | -5.5255 |
| 2,4,6-trichlorophenol | 0.024953 | -5.3246 |
| p1,p6-Bis5'-adenosylhexaphosphate | 0.031408 | -4.9927 |
| Clidinium | 0.033027 | -4.9202 |
| Molybdate | 0.037771 | -4.7266 |
| Sedoheptulose 1,7-bisphosphate | 0.055462 | -4.1724 |
| beta-D-Glucosyl-N-oleoylsphingosine | 0.065227 | -3.9384 |
| 1R,2S,3R,4R,5S,6R,7S-1,3,4,5,7,8,9,10,10-Nonachlorotricyclo5.2.1.0,dec-8-ene | 0.087573 | -3.5134 |
| N-2,6-dichlorophenylmethylideneaminoguanidine | 0.11616 | -3.1058 |
| Guanosine 2',3'-cyclic phosphate | 0.11616 | -3.1058 |
| 4-2R,3S,4R,5R-5-6-amino-9H-purin-9-yl-4-hydroxy-3-phosphonooxyoxolan-2-ylmethoxyhydroxyphosphoryloxyhydroxyphosphoryloxy-N-2-2-heptanoylsulfanylethyl-C-hydroxycarbonimidoylethyl-2-hydroxy-3,3-dimethylbutanimidic acid | 0.12122 | -3.0443 |
| Iodide | 0.16151 | -2.6303 |
| Carbamoyl phosphate | 0.22764 | -2.1352 |
| Selenocystine | 0.30648 | -1.7061 |
| 1-Methyladenosine | 0.35458 | -1.4958 |
| PCp-160/00 | 0.39508 | -1.3398 |
| 2-3-docosanoyloxy-2-1Z,11Z-octadeca-1,11-dien-1-yloxypropyl phosphonatooxyethyltrimethylazanium | 0.40332 | -1.31 |
| Adrenorphin | 0.43217 | -1.2103 |
| N2-Succinyl-L-glutamic acid 5-semialdehyde | 0.46008 | -1.12 |
| 3-Mercaptolactic acid | 0.46121 | -1.1165 |
| Taurine | 0.46515 | -1.1042 |
| TG1619Z/200/2045Z,8Z,11Z,14Ziso6 | 2.0225 | 1.0161 |
| 2,3,5-Trichlorodienelactone | 2.0285 | 1.0204 |
| Pipobroman | 2.1004 | 1.0707 |
| 17Z-N-2S,3R,4E-1-2R,5S,6R-3,5-dihydroxy-6-hydroxymethyl-4-sulfooxyoxan-2-yloxy-3-hydroxyoctadec-4-en-2-ylhexacos-17-enimidic acid | 3.9121 | 1.968 |
| 3R-10-oxo-8-Azatricyclo5.3.1.0,undecan-5-yl 1H-indole-3-carboxylate | 4.1502 | 2.0532 |
| beta-D-Glucosyl-N-docosanoylsphingosine | 4.9345 | 2.3029 |
| 1-2-2-chloro-3-thienylmethoxy-2-2,4-dichlorophenylethylimidazole | 5.1254 | 2.3577 |
| 4-Acetyloxy-2-7-4,5-dihydroxy-3-3,4,5-trihydroxy-6-methyloxan-2-yloxyoxan-2-yloxy-2,6,6,10,16-pentamethyl-18-2-methylprop-1-en-1-yl-19,21-dioxahexacyclo18.2.1.0,.0,.0,.0,tricosan-16-yloxy-5-hydroxy-6-methyloxan-3-yl acetate | 6.6971 | 2.7435 |
| Trichloroacetic acid | 6.7434 | 2.7535 |
| Chlordecone | 6.7434 | 2.7535 |
| PC220/00 | 7.3846 | 2.8845 |
| Dimethyl trisulfide | 7.9995 | 2.9999 |
| 17Z-N-4Z-1-2R,3R,4R,5R,6R-3,4-dihydroxy-6-hydroxymethyl-5-2R,3R,4S,5R,6R-3,4,5-trihydroxy-6-hydroxymethyloxan-2-yloxyoxan-2-yloxy-3-hydroxyoctadec-4-en-2-ylhexacos-17-enimidic acid | 8.3033 | 3.0537 |
| Naled | 8.4446 | 3.078 |
| 2-Aminoethoxy2R-3-13Z-docos-13-enoyloxy-2-1Z-octadec-1-en-1-yloxypropoxyphosphinic acid | 8.9785 | 3.1665 |
| CerD181/250 | 12.662 | 3.6624 |
| 4-2R,3S,4R,5R-5-6-amino-9H-purin-9-yl-4-hydroxy-3-phosphonooxyoxolan-2-ylmethoxyhydroxyphosphoryloxyhydroxyphosphoryloxy-N-2-2-3Z,6Z-dodeca-3,6-dienoylsulfanylethyl-C-hydroxycarbonimidoylethyl-2-hydroxy-3,3-dimethylbutanimidic acid | 12.832 | 3.6817 |
| Malonic acid | 15.869 | 3.9882 |
| D-Cysteine | 15.967 | 3.997 |
| CerD181/24115Z | 19.21 | 4.2638 |
| 2,4,6-Tribromophenol | 20.849 | 4.3819 |
| DITP | 22.152 | 4.4694 |
| Nadp | 22.69 | 4.504 |
| e-3-phenyl-1-3,4,5-trihydroxy-6-hydroxymethyloxan-2-ylsulfanylpropylideneaminooxysulfonic acid | 30.103 | 4.9118 |
| Thiabendazole | 36.237 | 5.1794 |
| 5-Diphosphoinositol pentakisphosphate | 39.399 | 5.3001 |
| 2,2,2-Trichloroethanol | 60.26 | 5.9131 |
| Trimethyl2-2-1Z-octadec-1-en-1-yloxy-3-tetracosanoyloxypropyl phosphonatooxyethylazanium | 64.132 | 6.003 |
| CerD181/120 | 131.26 | 7.0363 |
| Mitomycin C | 206.56 | 7.6904 |

**Supplementary Table 4:** Significant features identified by t- test and volcano plot in seminal plasma of astheno-oligzoospermia bulls

| Compound name | Retention time | Mass/charge | t-test | **t-** test ( p value) | FC | log2(FC) | p. adjusted at 0.05 |
| --- | --- | --- | --- | --- | --- | --- | --- |
| Tetradecanoyl-CoA | 1.47 | 324.72 | -7.3636 | 0.001812 | 0.018709 | -5.7401 | 0.009061 |
| Malonic acid | 0.15 | 102.96 | 10.348 | 0.000492 | 11.178 | 3.4826 | 0.007384 |
| 5-Diphosphoinositol pentakisphosphate | 1.54 | 368.88 | 8.502 | 0.00105 | 2.746 | 1.4574 | 0.007872 |
