# Supplementary material for: Deep Metabolomic Profiling Reveals Alterations in Fatty Acid Synthesis and Ketone Body Degradations in Spermatozoa and Seminal Plasma of Astheno-Oligozoospermic Bulls
Source: Front Vet Sci. 2022 Jan 11;8:755560. doi: 10.3389/fvets.2021.755560 (PMC8787163; doi:10.3389/fvets.2021.755560)
Supplement: Supplementary file 2 [file Data_Sheet_2.docx]

**Supplementary (figures)**

**Manuscript Title:** Deep metabolomic profiling reveals alterations in fatty acid synthesis and ketone body degradations in spermatozoa and seminal plasma of astheno-oligozoospermic bulls

**Authors:** Mohua Dasgupta, Arumugam Kumaresan^*^, Kaustubh Kishor Saraf, Pradeep Nag, Manish Kumar Sinha, Muhammad Aslam M. K, Gayathree Karthikkeyan, T. S. Keshava Prasad, Prashant Kumar Modi, Tirtha Kumar Datta, Kerekoppa Ramesha, Ayyasamy Manimaran, Sakthivel Jeyakumar


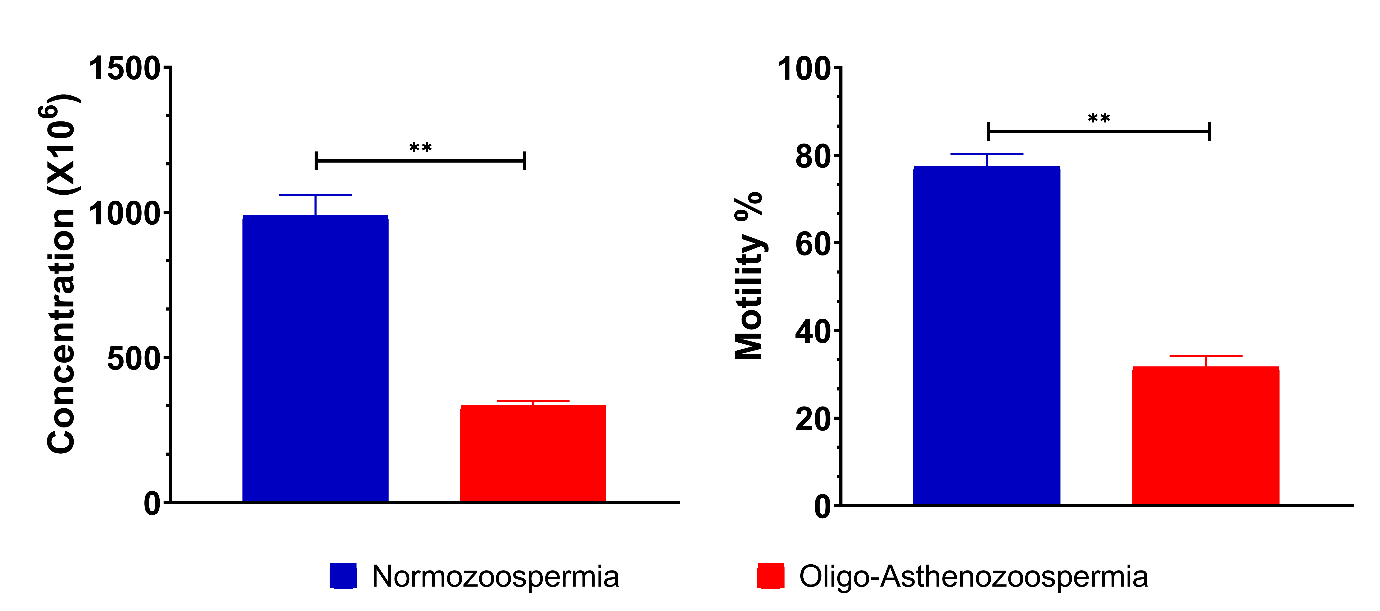


**Supplementary Figure 1:** Sperm concentration and motility in normozoospermic and Oligoasthenozoospermic bulls. Both sperm motility and concentration significantly (P<0.01) differed between normozoospermic and Oligoasthenozoospermic bulls.


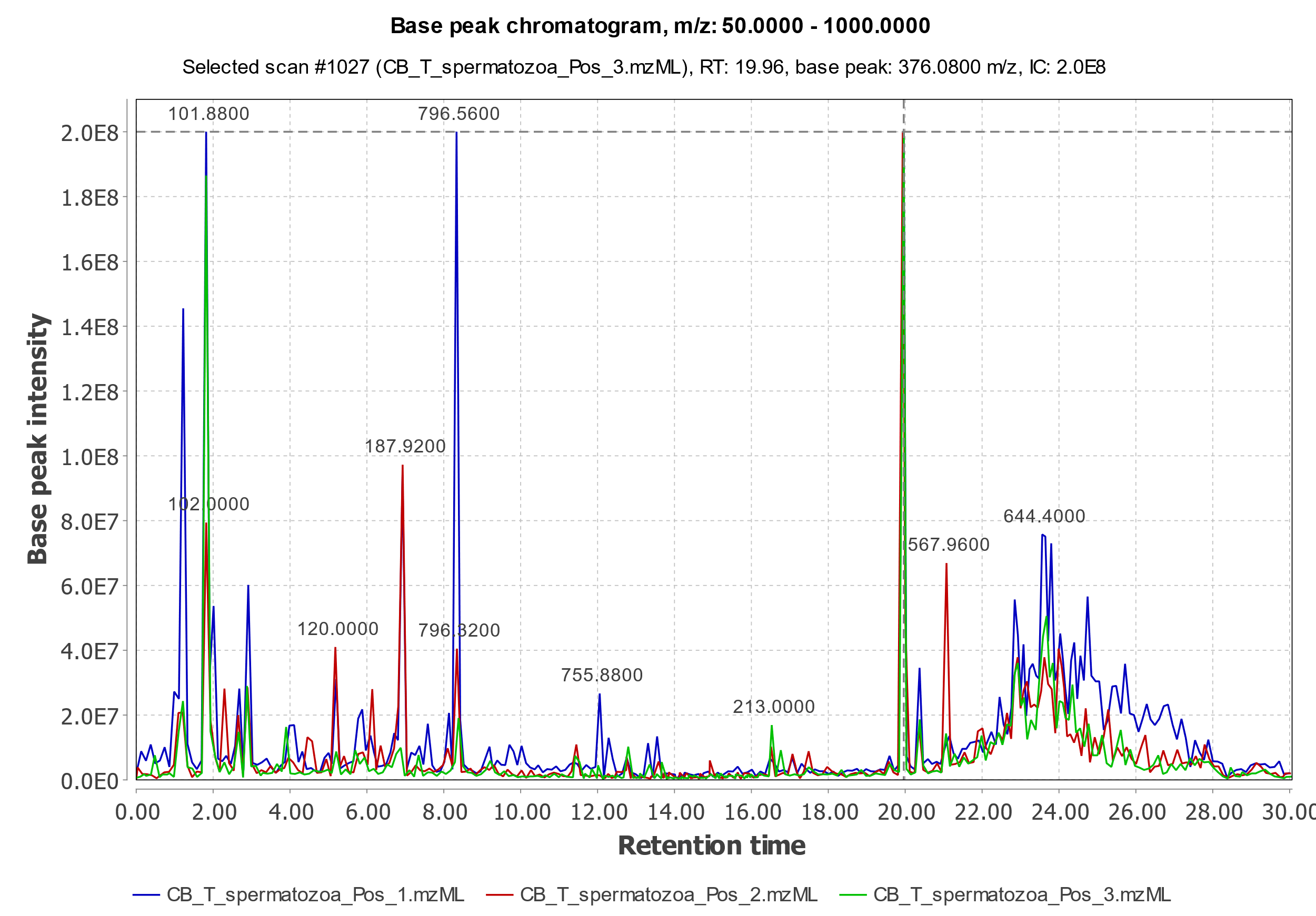


**A**


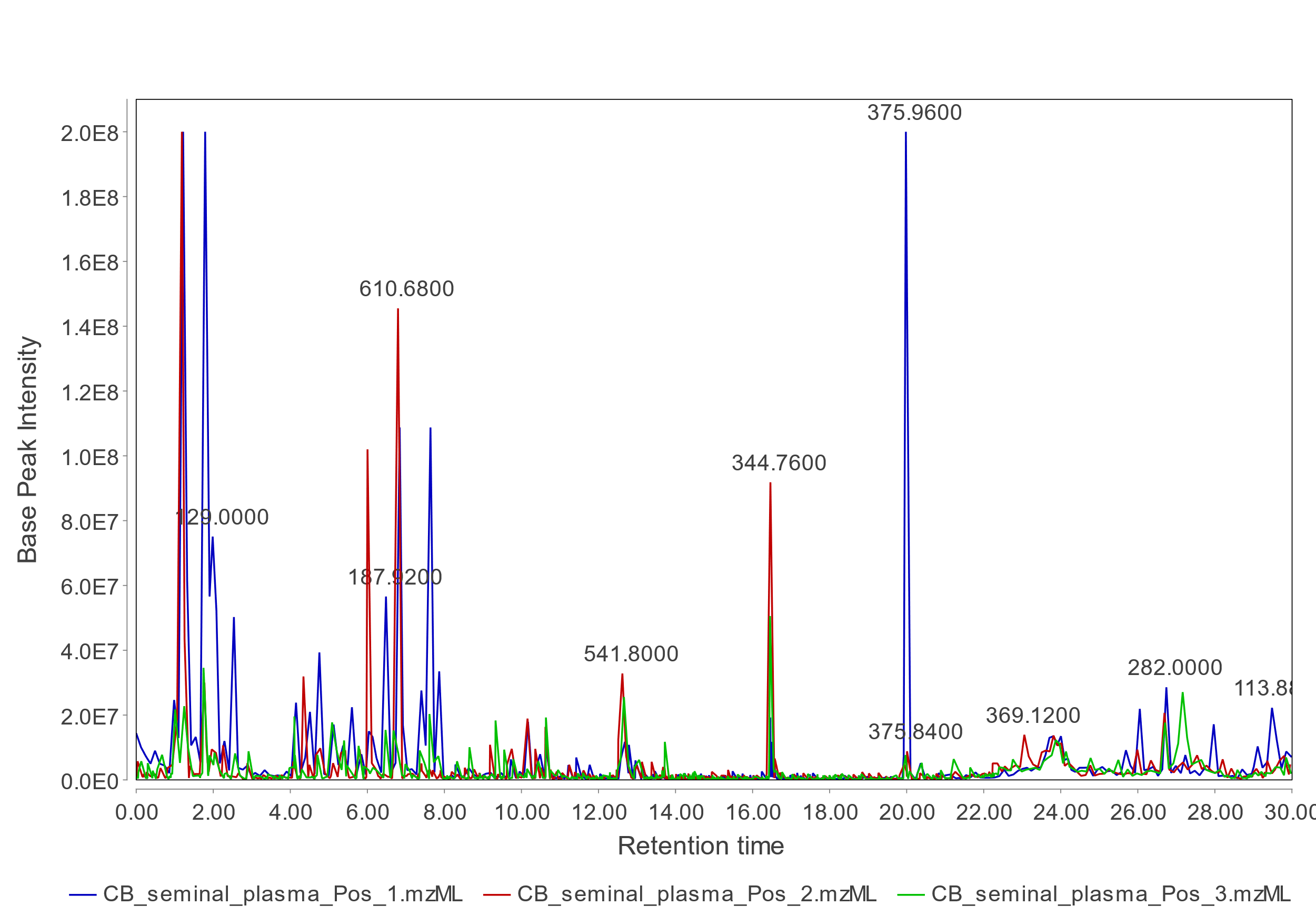


**B**

**Supplementary Figure 2(A, B) :** LC/MS MS chromatogram of some endogenous metabolites of both the group


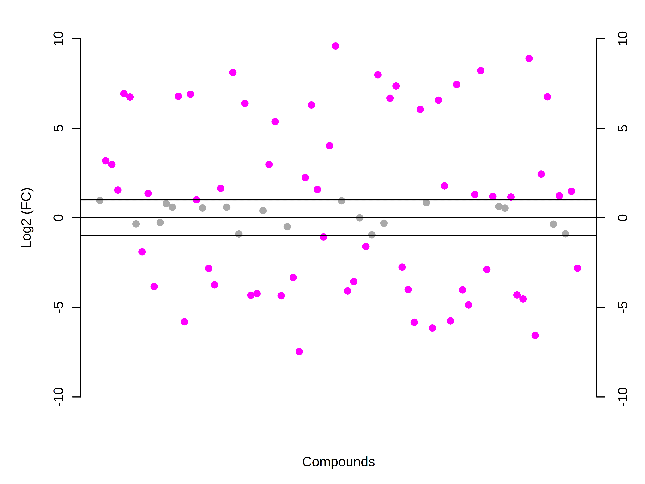

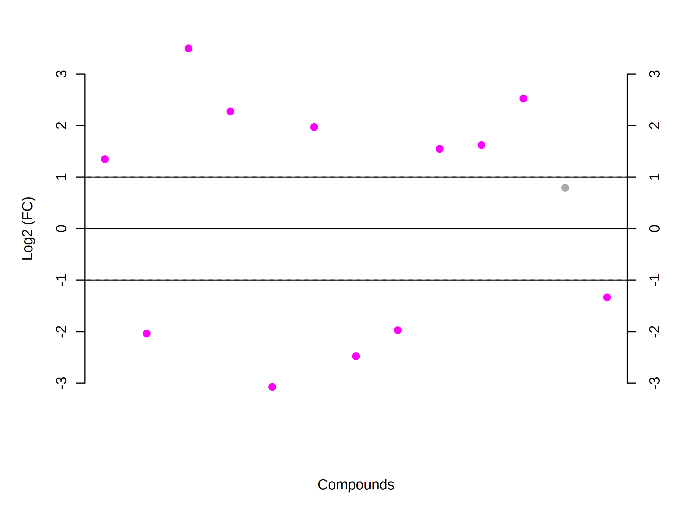


**A**

**B**

**Supplementary Figure 3(A, B):** Graphical presentation of top 50 metabolites and selected features of spermatozoa based on fold change analysis with threshold 2


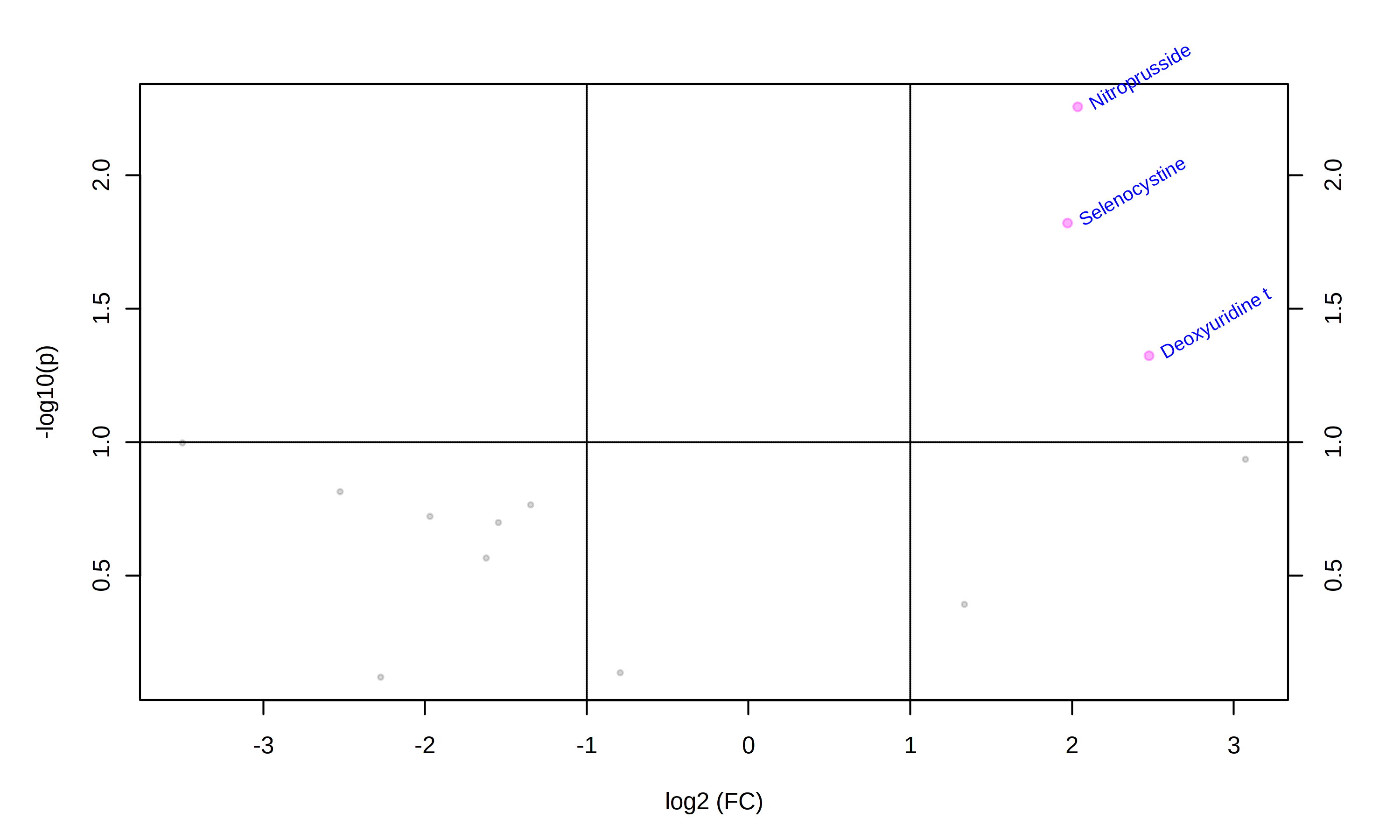


**Supplementary Figure 4:** Graphical representation of significant features identified by volcano plot in spermatozoa of astheno-oligozoospermia bulls

**A**

**B**


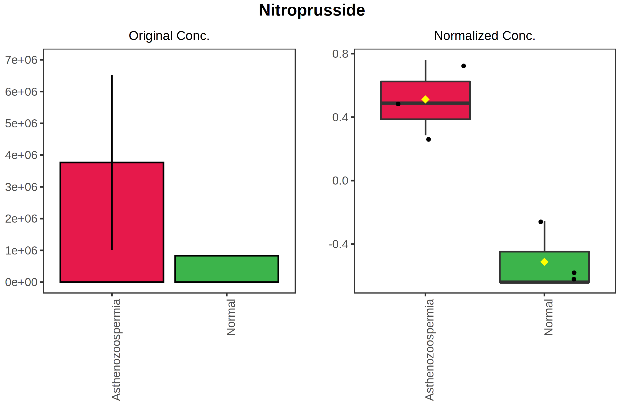

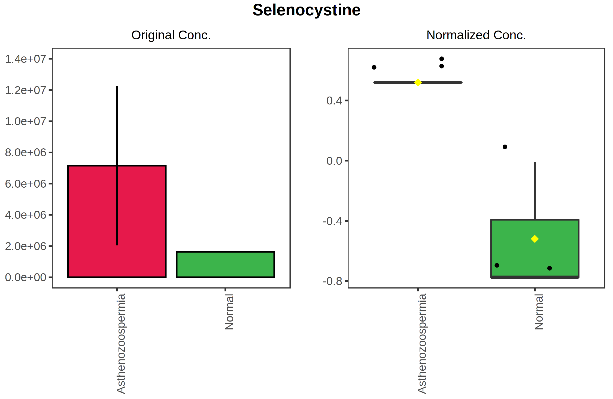


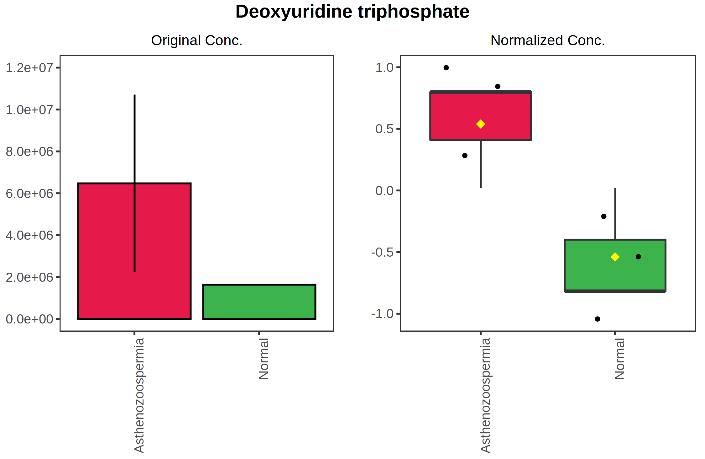


**C**

**Supplementary Figure 5 (A, B, C):** Box plots of abundance ratio of sperm metabolites above VIP score 1


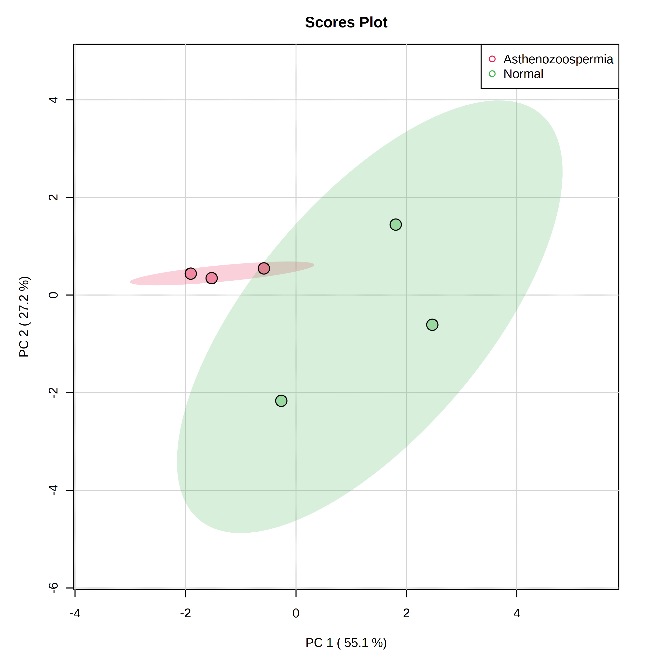

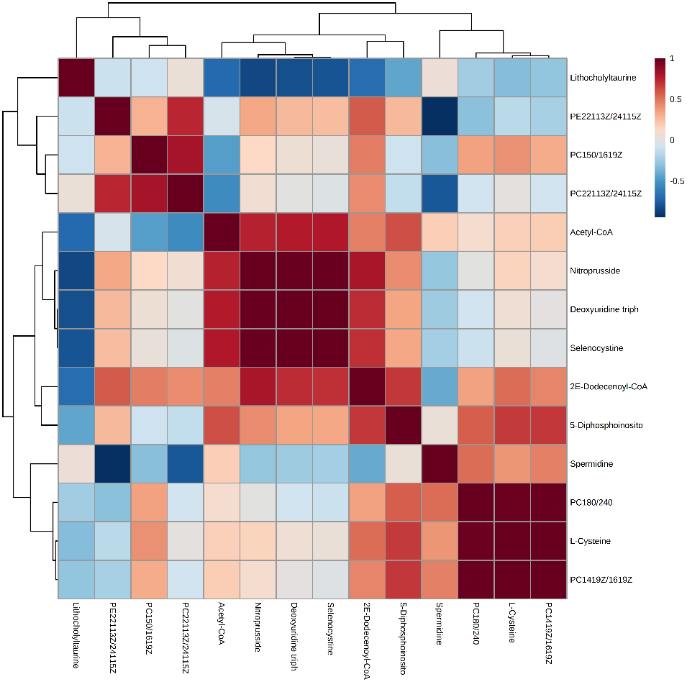


**B**

**A**

**Supplementary Figure 6 (A, B):** Principal component analysis and correlation plot analysis of significant sperm metabolites


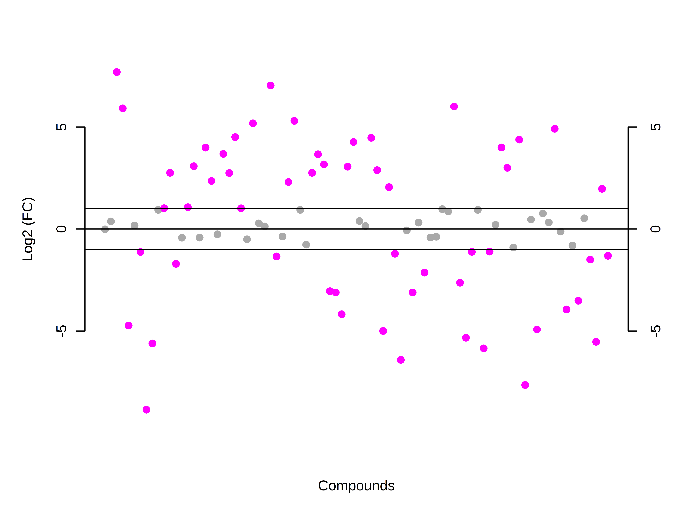

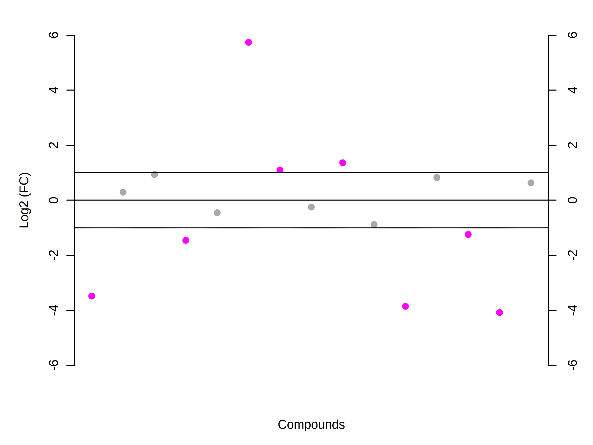


**A**

**B**

**Supplementary Figure 7(A, B):** Graphical presentation of top 50 and selected metabolites of seminal plasma based on fold change analysis with threshold 2


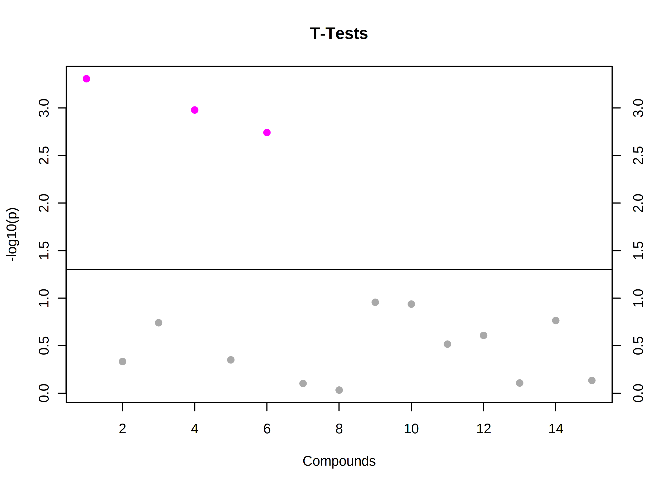

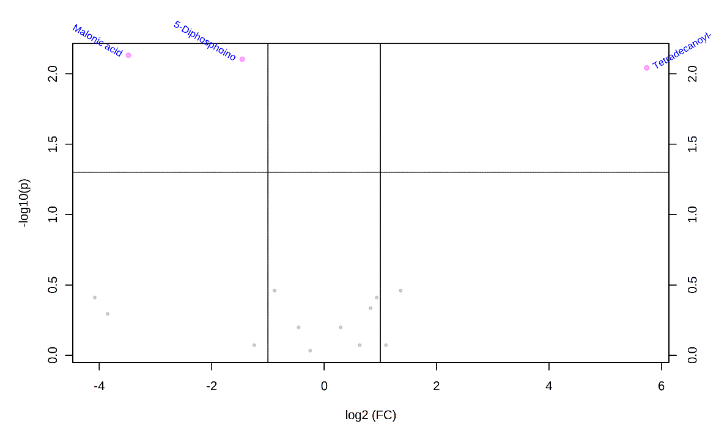


**A**

**B**

**Supplementary Figure 8(A, B):** Graphical representation of significant features identified by t- test & volcano plot seminal plasma of astheno-oligozoospermia bulls


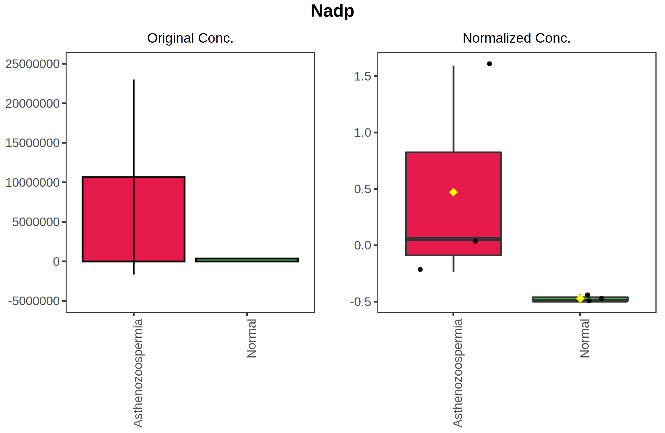

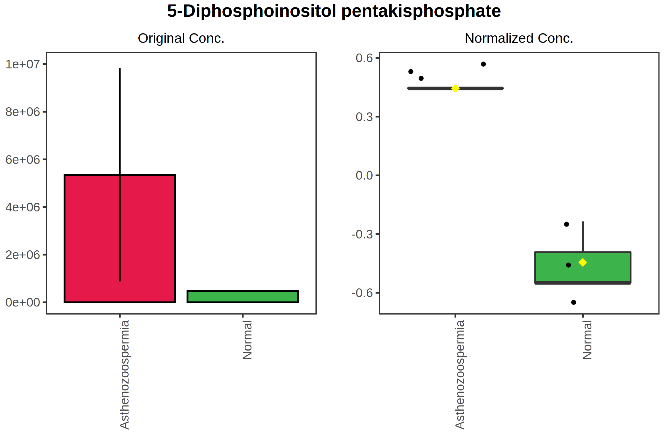

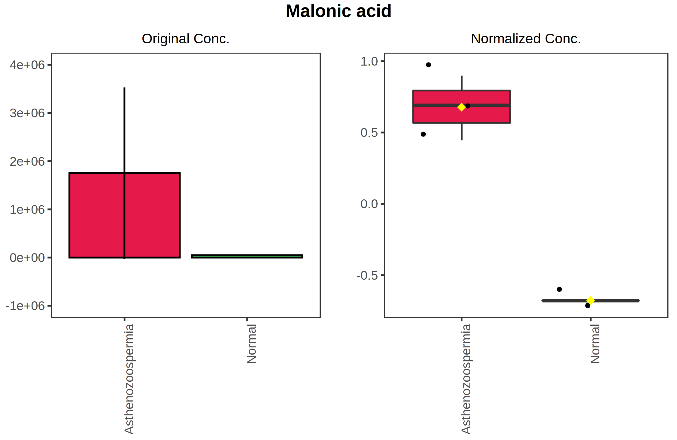

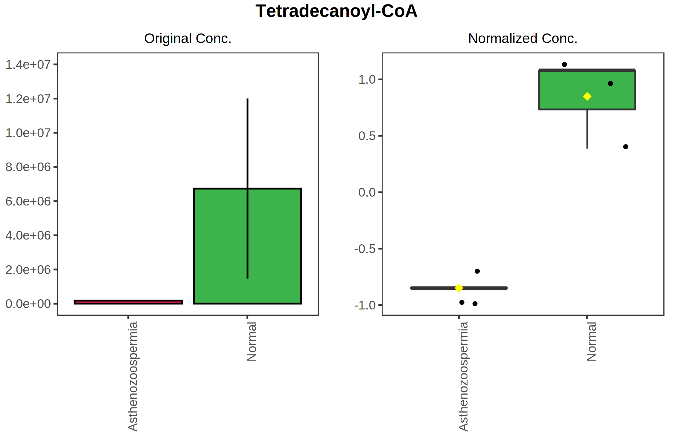

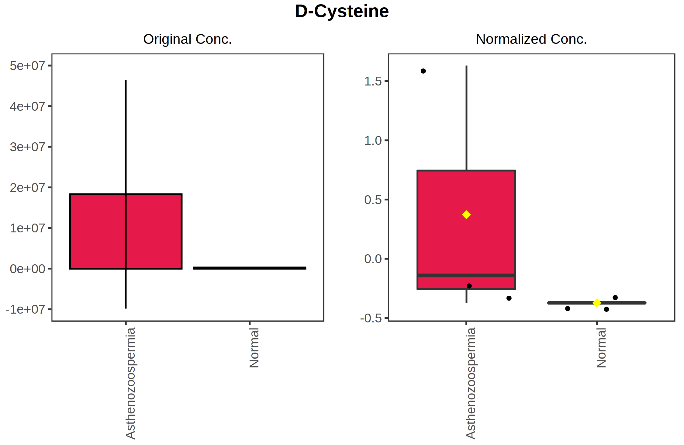


**B**

**E**

**D**

**C**

**A**

**Supplementary Figure 9(A, B, C, D, E):** Abundance of significant metabolites of seminal plasma, based on VIP score ≥ 1


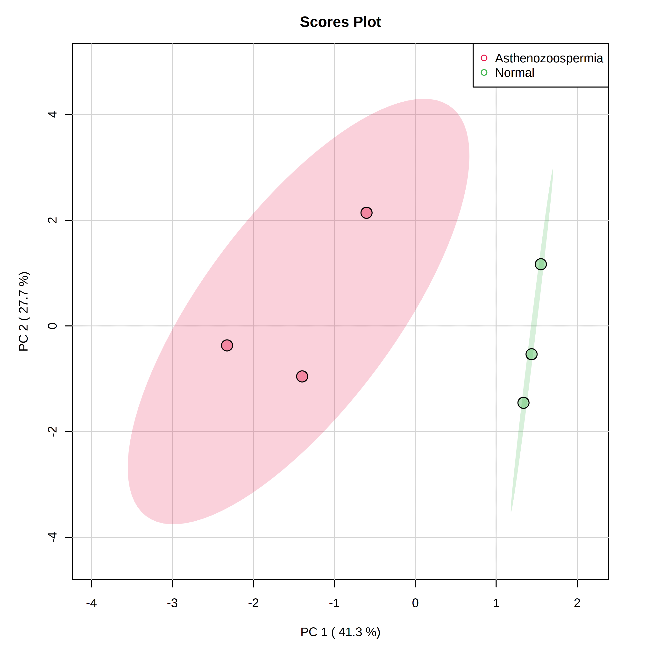

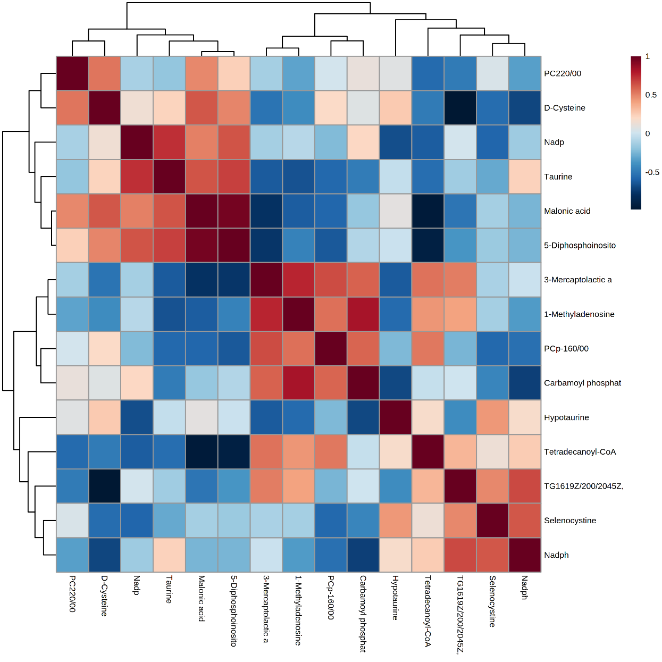


**B**

**A**

**Supplementary Figure 10 (A, B):** Principal component analysis and correlation plot analysis of significant seminal plasma metabolites


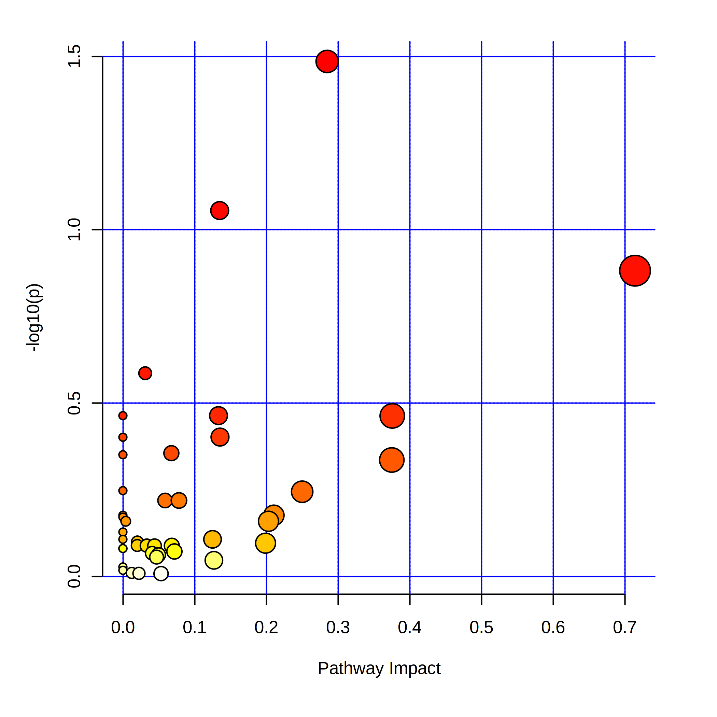

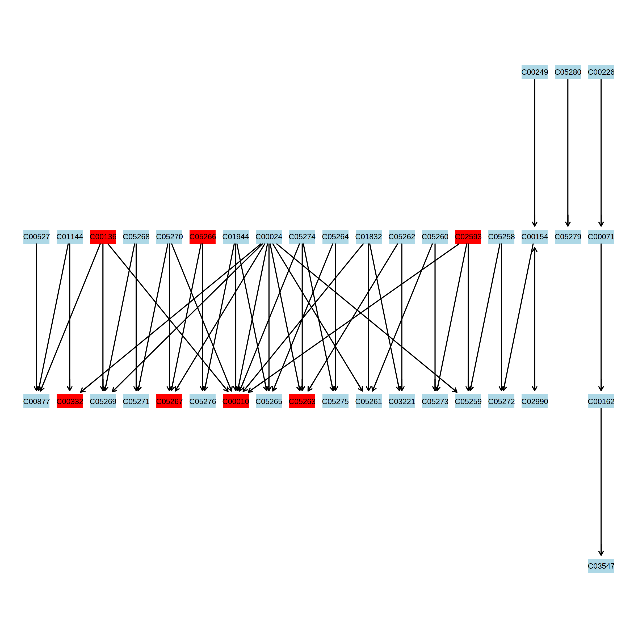


**A**

**Supplementary Figure 11 A:** The metabolome view of the graphical output of significant pathways of spermatozoa of astheno-oligozoospermia bulls


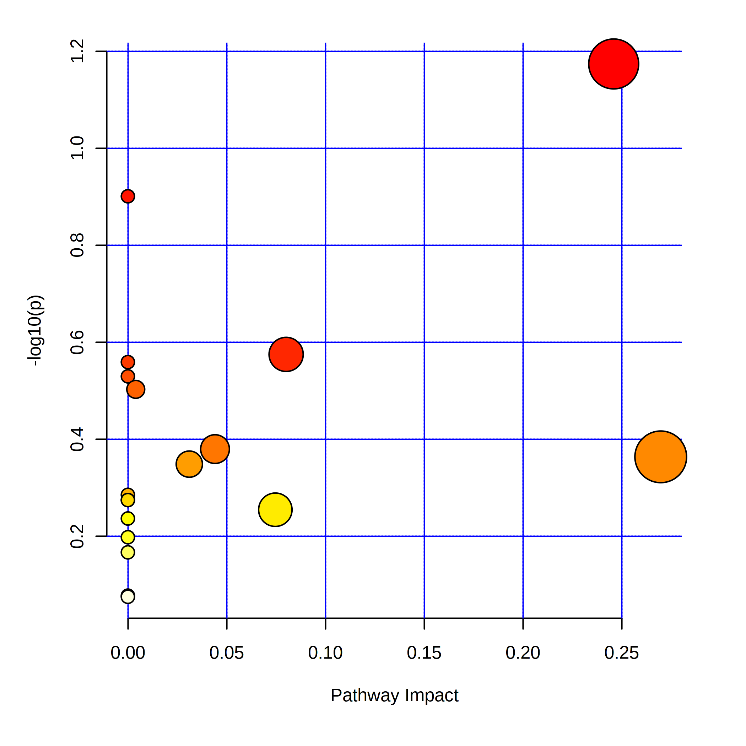

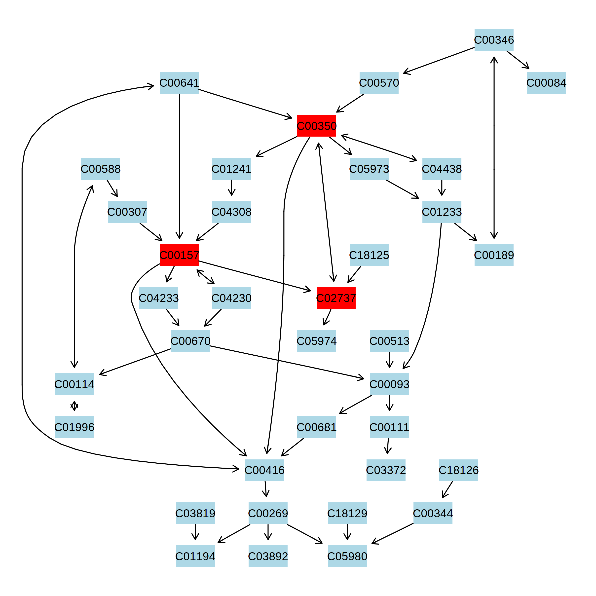


**B**

**Supplementary Figure 11 B:** The metabolome view of the graphical output of significant pathways of spermatozoa of normozoospermia bulls


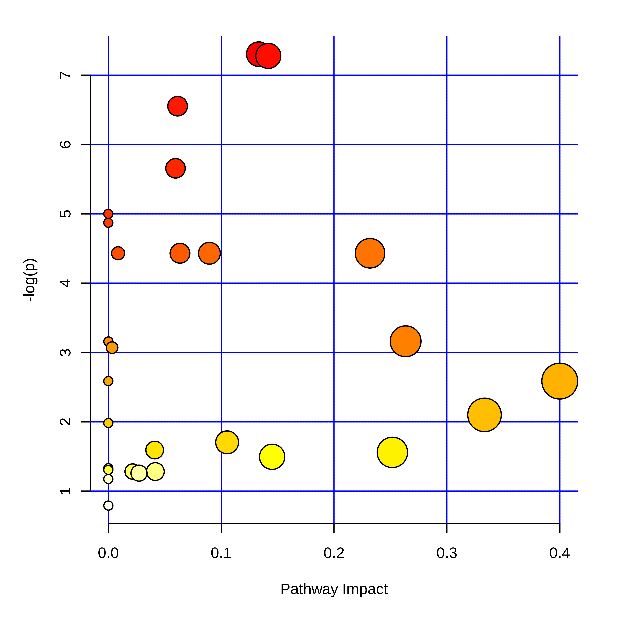

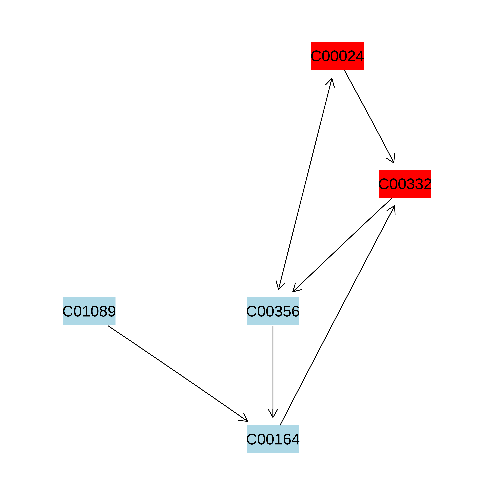


**Supplementary Figure 12 A:** The metabolome view of the graphical output of significant pathways of seminal plasma of astheno-oligozoospermia bulls


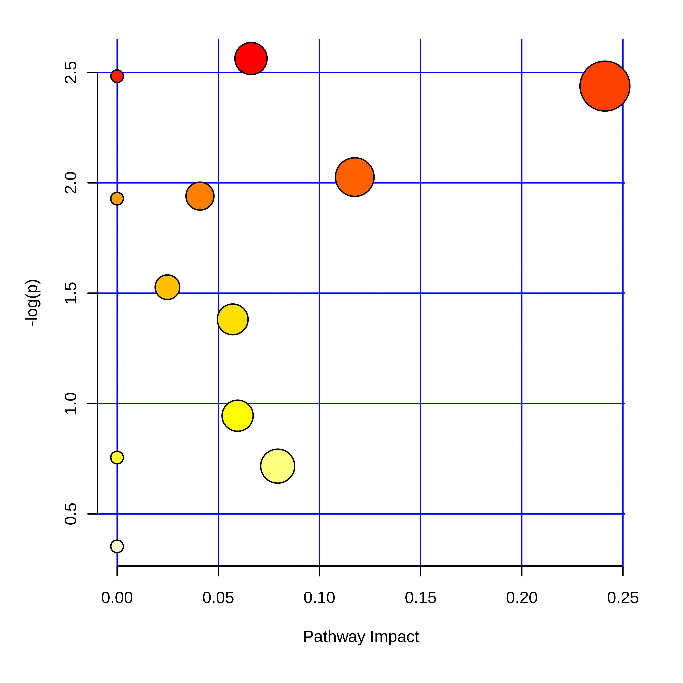

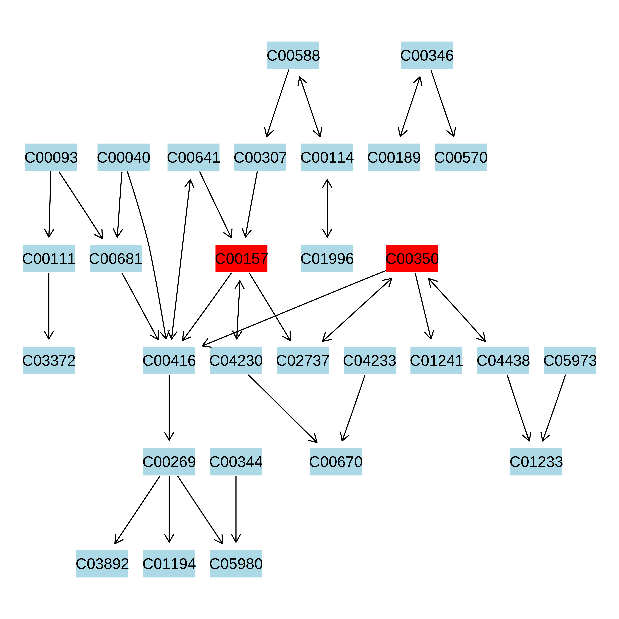


**Supplementary Figure 12 B:** The metabolome view of the graphical output of significant pathways of seminal plasma of normozoospermia bulls
